# Supplementary material for: Genomic basis of the giga-chromosomes and giga-genome of tree peony Paeonia ostii
Source: Nat Commun. 2022 Nov 28;13:7328. doi: 10.1038/s41467-022-35063-1 (PMC9705720; doi:10.1038/s41467-022-35063-1)
Supplement: Supplementary file 1 — Supplementary Information file [file 41467_2022_35063_MOESM1_ESM.pdf]

Genomic basis of the giga-chromosomes and giga-genome of tree peony *Paeonia ostii*

Yuan *et al.*

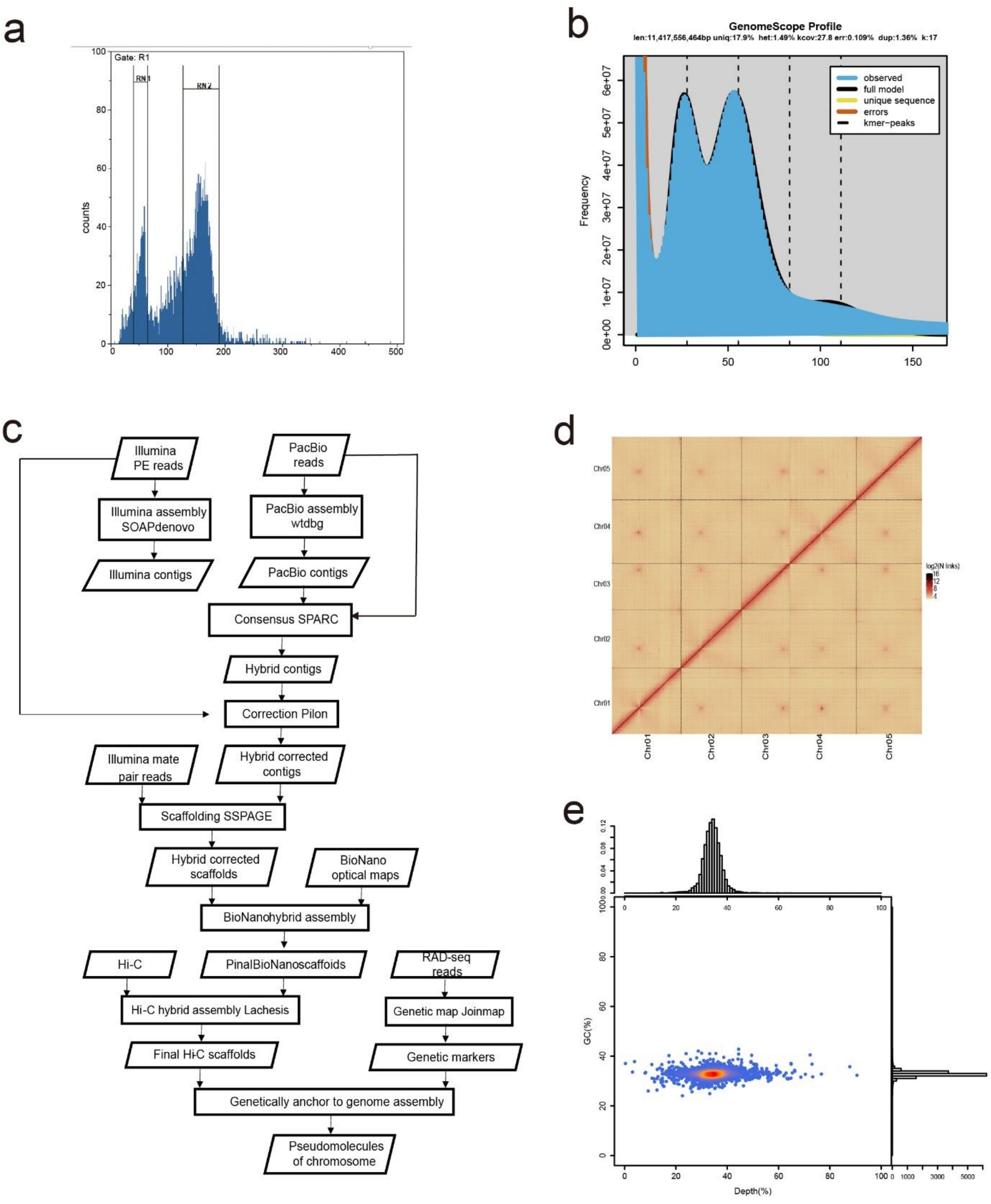

Supplementary Fig. 1. Genome assembly of *P. ostii*. a, Estimation of *P. ostii* genome size by flow cytometry analysis. **b,** K-mer analysis of *P. ostii* genome by GenomeScope. The heterozygosity was estimated to be 1.5%. **c,** The genome assembly pipeline of *P. ostii*by a combination of sequencing data. **d,** Overview of the Hi-C heatmap of the *P. ostii* genome showing genome-wide all-by-all interactions with in 750 kb. **e,** GC ratio in relation to sequencing depth. Source data are provided as a Source Data file.

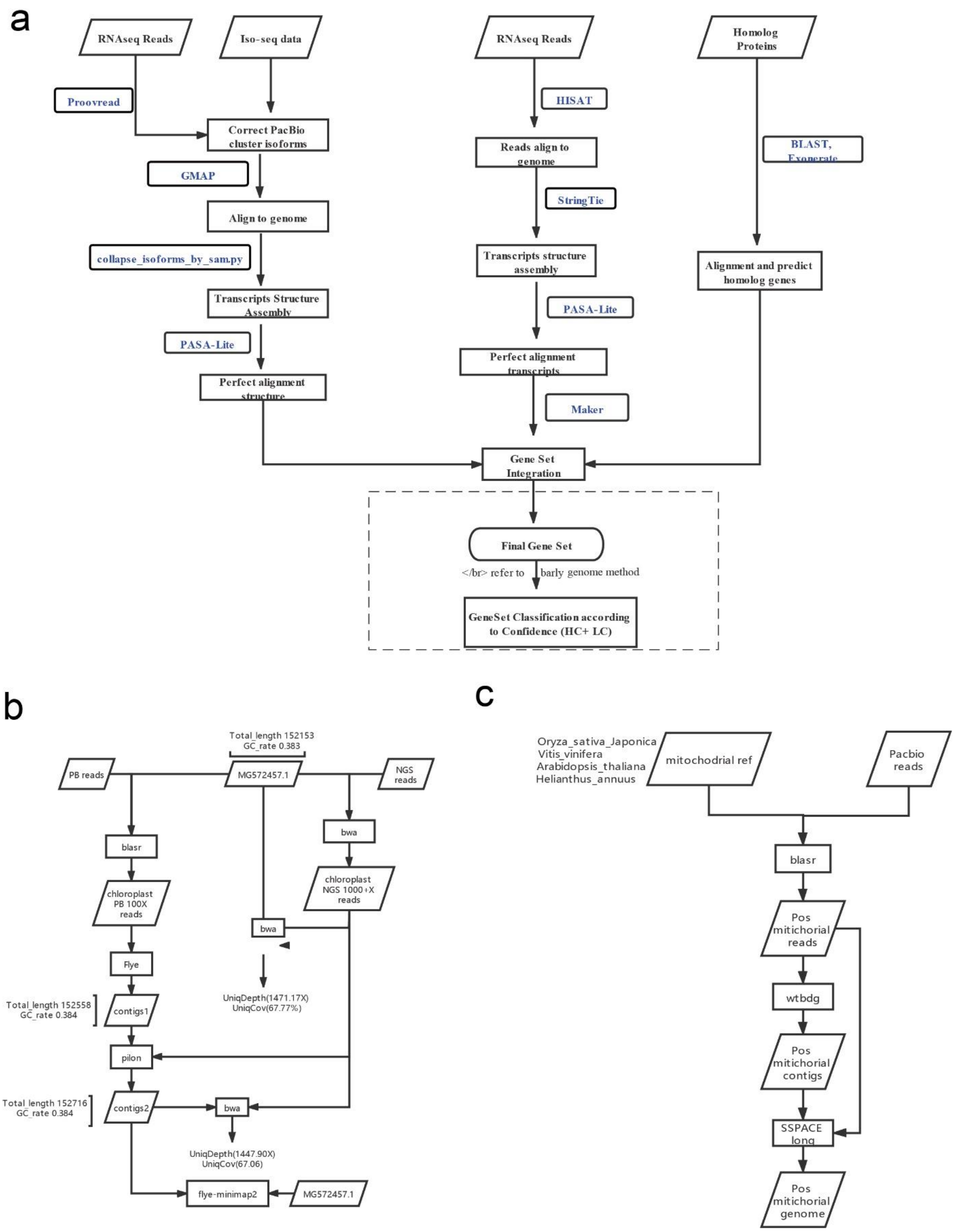

Supplementary Fig. 2. Genome annotation and organelle assembly of *P. ostii.* a, The annotation pipeline of the tree peony genome. **b,** The assembly pipeline of the*P. ostii* chloroplast. **c**, The assembly pipeline of *P. ostii* mitochondrial sequence.

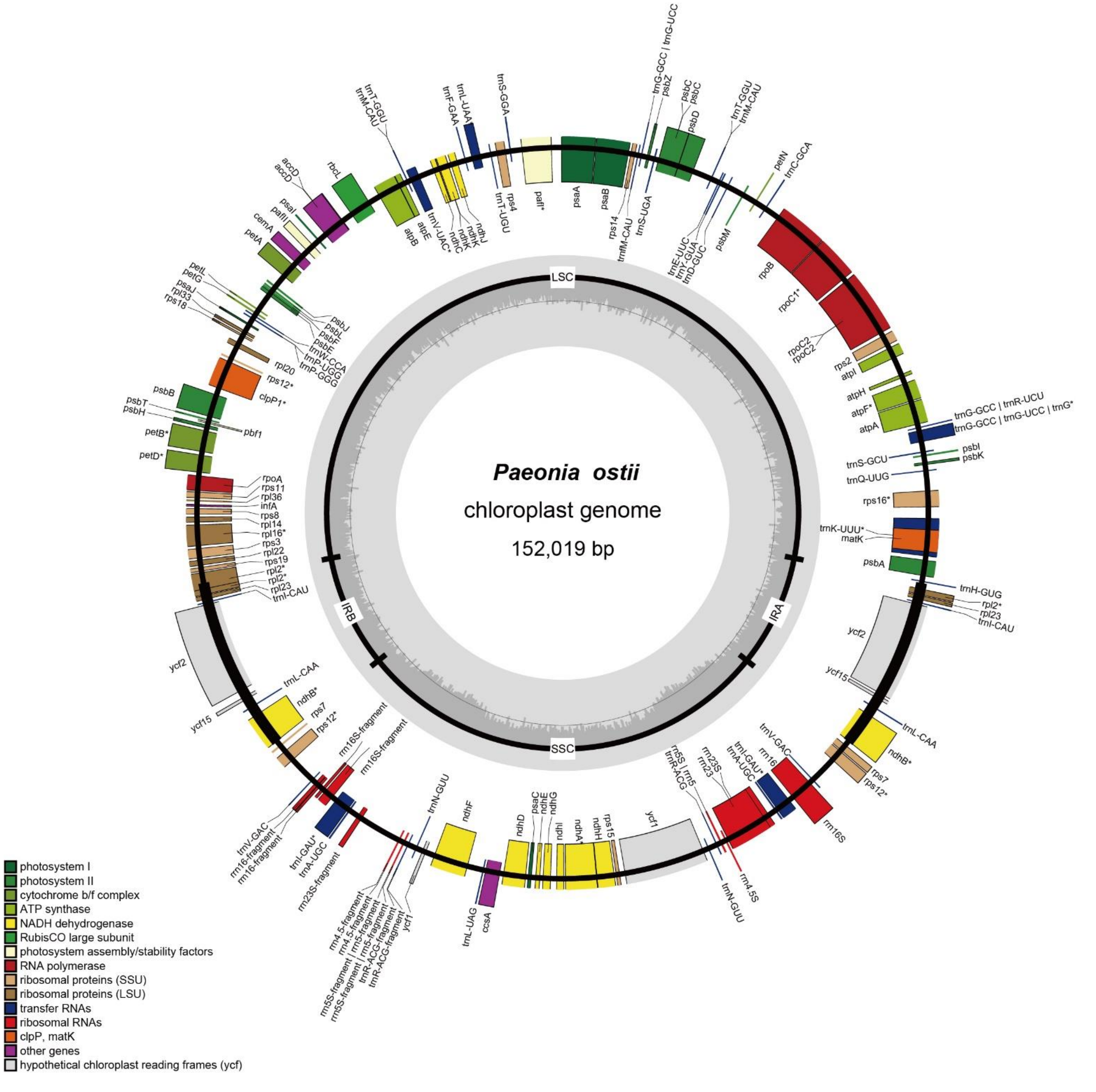

Supplementary Fig. 3. Circos view of *P. ostii* chloroplast. Source data are provided as a Source Data file.

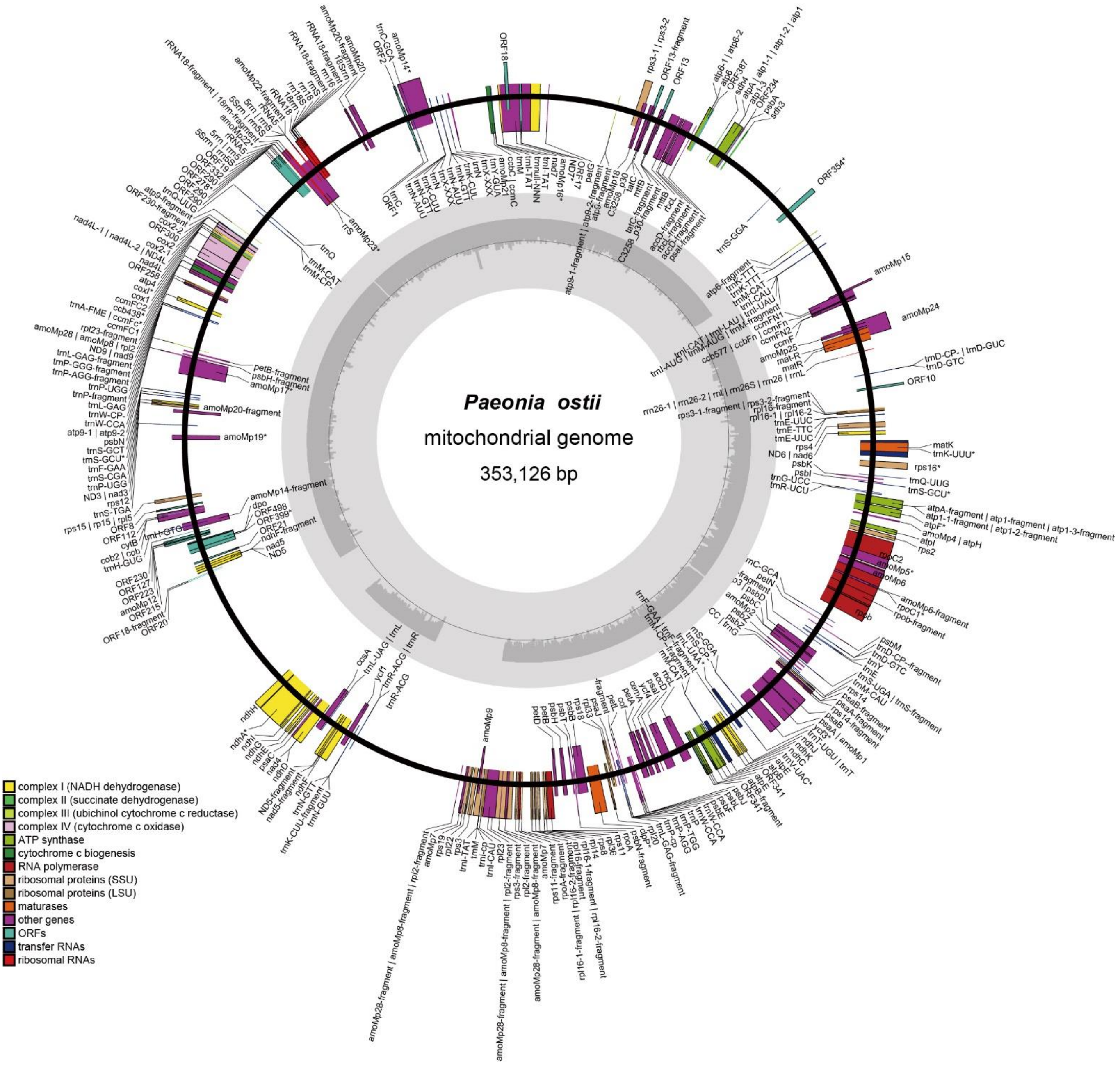

Supplementary Fig. 4. Circos view of the *P. ostii* mitochondrial genome. Source data are provided as a Source Data file.

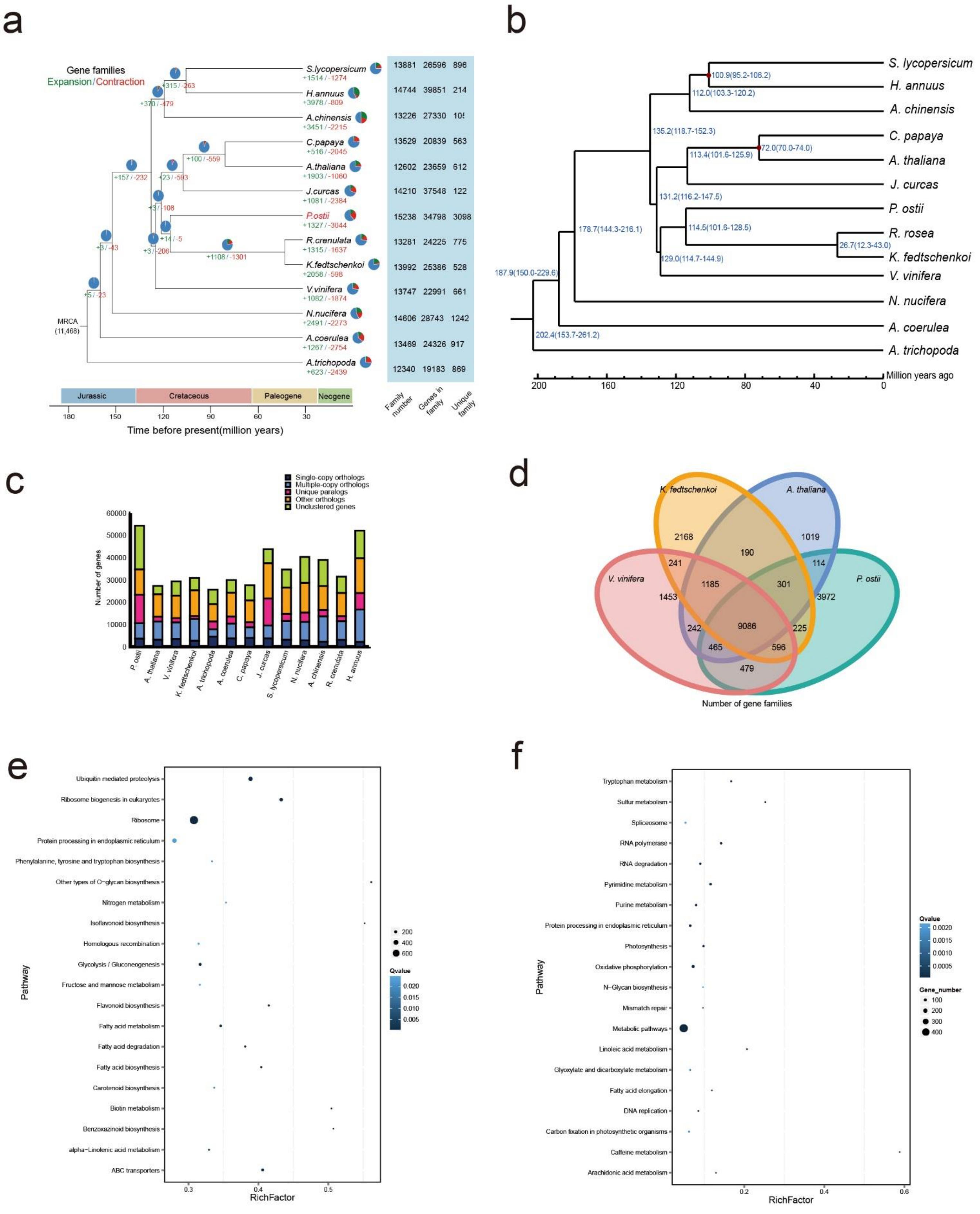

a
Gene families
Expansion/Contraction
S.lycopersicum +1514 / -1274
H.annuus +3978 / -809
A.chinensis +3451 / -2215
C.papaya +516 / -2045
A.thaliana +1903 / -1060
J.curcas +1081 / -2384
P.ostii +1327 / -3044
R.crenulata +1315 / -1637
K.fedtschenkoi +2058 / -598
V.vinifera +1082 / -1874
N.nucifera +2491 / -2273
A.coerulea +1267 / -2754
A.trichopoda +623 / -2439
MRCA (11,468)
13881 26596 896
14744 39851 214
13226 27330
13529 20839 563
12602 23659 612
14210 37548 122
15238 34798 3098
13281 24225 775
13992 25386 528
13747 22991 661
14606 28743 1242
13469 24326 917
12340 19183 869
Family number
Genes in family
Unique family
Jurassic
Cretaceous
Paleogene
Neogene
180 150 120 90 60 30
Time before present(million years)
b
S. lycopersicum
H. annuus
A. chinensis
C. papaya
A. thaliana
J. curcas
P. ostii
R. rosea
K. fedtschenkoi
V. vinifera
N. nucifera
A. coerulea
A. trichopoda
100.9(95.2-106.2)
112.0(103.3-120.2)
135.2(118.7-152.3)
72.0(70.0-74.0)
113.4(101.6-125.9)
131.2(116.2-147.5)
114.5(101.6-128.5)
26.7(12.3-43.0)
129.0(114.7-144.9)
178.7(144.3-216.1)
187.9(150.0-229.6)
202.4(153.7-261.2)
200 160 120 80 40 0
Million years ago
c
Single-copy orthologs
Multiple-copy orthologs
Unique paralogs
Other orthologs
Unclustered genes
Number of genes
60000 50000 40000 30000 20000 10000 0
P. ostii
A. thaliana
V. vinifera
K. fedtschenkoi
A. trichopoda
A. coerulea
C. papaya
J. curcas
S. lycopersicum
N. nucifera
A. chinensis
R. crenulata
H. annuus
d
K. fedtschenkoi
A. thaliana
V. vinifera
P. ostii
2168
1019
190
241
114
1453
1185
301
3972
242
9086
225
465
596
479
Number of gene families
e
Ubiquitin mediated proteolysis
Ribosome biogenesis in eukaryotes
Ribosome
Protein processing in endoplasmic reticulum
Phenylalanine, tyrosine and tryptophan biosynthesis
Other types of O-glycan biosynthesis
Nitrogen metabolism
Isoflavonoid biosynthesis
Homologous recombination
Glycolysis / Gluconeogenesis
Fructose and mannose metabolism
Flavonoid biosynthesis
Fatty acid metabolism
Fatty acid degradation
Fatty acid biosynthesis
Carotenoid biosynthesis
Biotin metabolism
Benzoxazinoid biosynthesis
alpha-Linolenic acid metabolism
ABC transporters
Pathway
RichFactor
0.3 0.4 0.5
200
400
600
Qvalue
0.020
0.015
0.010
0.005
f
Tryptophan metabolism
Sulfur metabolism
Spliceosome
RNA polymerase
RNA degradation
Pyrimidine metabolism
Purine metabolism
Protein processing in endoplasmic reticulum
Photosynthesis
Oxidative phosphorylation
N-Glycan biosynthesis
Mismatch repair
Metabolic pathways
Linoleic acid metabolism
Glyoxylate and dicarboxylate metabolism
Fatty acid elongation
DNA replication
Carbon fixation in photosynthetic organisms
Caffeine metabolism
Arachidonic acid metabolism
Pathway
RichFactor
0.2 0.4 0.6
Qvalue
0.0020
0.0015
0.0010
0.0005
Gene_number
100
200
300
400

Supplementary Fig. 5. Gene families of *P. ostii.* **a,** Phylogenetic relationships and gene family expansion/contraction on each evolutionary branch. A total of 1327 gene families were substantially expanded, and 3044 gene families were contracted, in peony compared with other plant genomes. The corresponding proportions among the total changes are shown as pie charts. Blue portions of the pie charts represent conserved gene families. Genomes of *Amborella trichopoda, Aquilegia coerulea, Carica papaya, Vitis vinifera, Solanum lycopersicum, Nelumbo nucifera, Jatropha curcas, Actinidia chinensis, Kalanchoe fedtschenkoi, Helianthus annuus* and *Rhodiola crenulate* were used for the evolutionary analysis. **b,** Multispecies coalescent tree of their chloroplast genes. **c,** Gene numbers in each gene family category in 13 land plant species. **d,** Venn diagram showing shared orthologous groups among tree peony, grape, *K. fedtschenkoi* and *A. thaliana*. Numbers in circles indicate the number of genes in each cluster. **e,** KEGG pathway functional enrichment of tree peony unique gene families. The statistics for the top 20 pathways are shown. Q values represent the significance of enrichment. Circles indicate the target genes, and the size is proportional to the number of genes. **f,** The KEGG pathway functional enrichment of tree peony showed significant results related to expanded gene families. The statistics for the top 20 pathways are shown. Q values represent the significance of enrichment. Circles indicate the target genes, and the size is proportional to the number of genes. Source data are provided as a Source Data file.

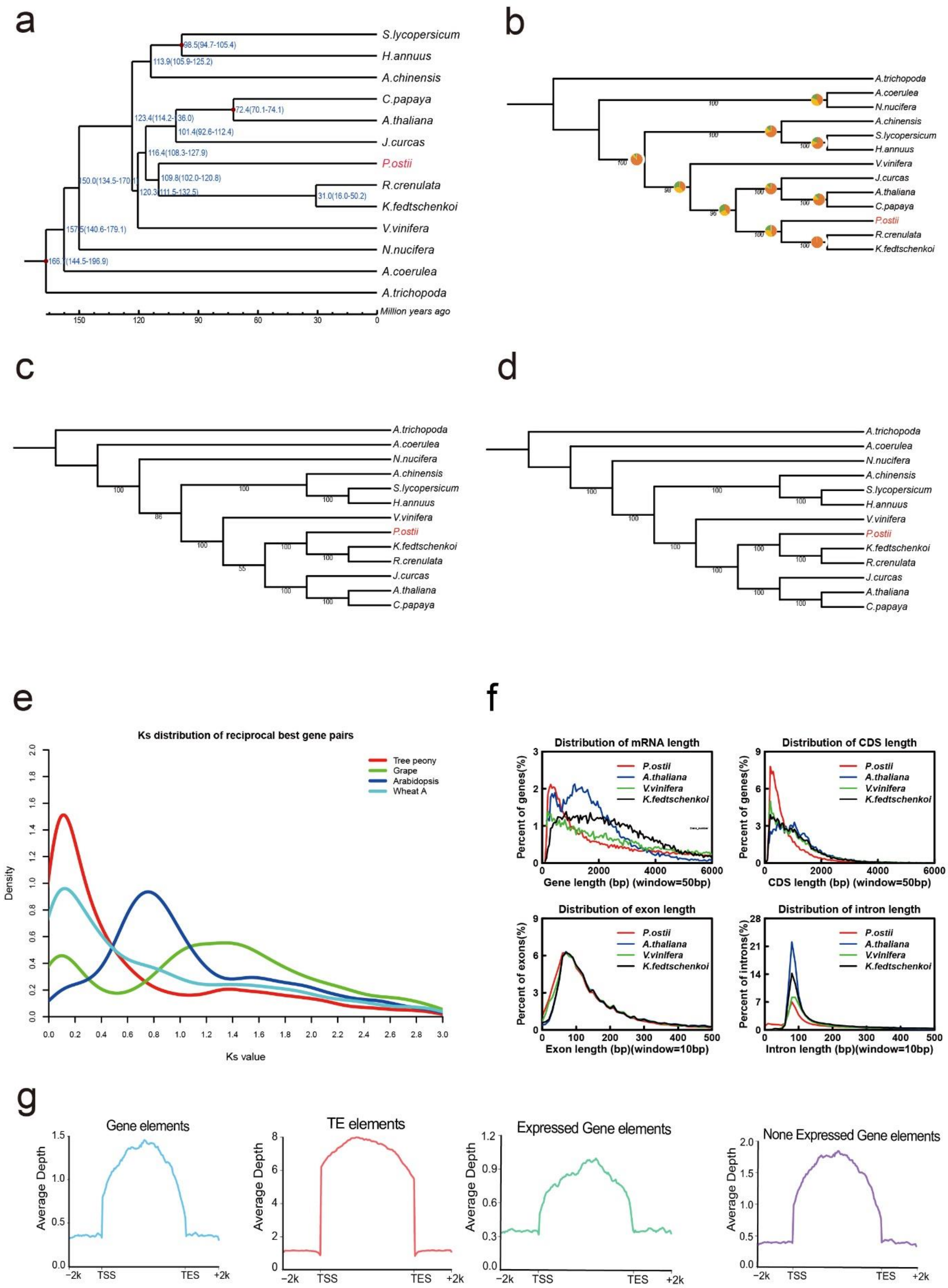
a
S.lycopersicum
H.annuus
A.chinensis
C.papaya
A.thaliana
J.curcas
P.ostii
R.crenulata
K.fedtschenkoi
V.vinifera
N.nucifera
A.coerulea
A.trichopoda
98.5(94.7-105.4)
113.9(105.9-125.2)
72.4(70.1-74.1)
101.4(92.6-112.4)
116.4(108.3-127.9)
109.8(102.0-120.8)
31.0(16.0-50.2)
Million years ago
150
120
90
60
30
0
b
c
d
e
Ks distribution of reciprocal best gene pairs
Tree peony
Grape
Arabidopsis
Wheat A
Density
Ks value
f
Distribution of mRNA length
Distribution of CDS length
Distribution of exon length
Distribution of intron length
P.ostii
A.thaliana
V.vinifera
K.fedtschenkoi
Percent of genes(%)
Percent of exons(%)
Percent of introns(%)
Gene length (bp) (window=50bp)
CDS length (bp) (window=50bp)
Exon length (bp)(window=10bp)
Intron length (bp)(window=10bp)
g
Gene elements
TE elements
Expressed Gene elements
None Expressed Gene elements
Average Depth
−2k
TSS
TES
+2k

Supplementary Fig. 6. Phylogenetics of *P. ostii.* **a,** Estimation of *P. ostii* divergence time. The blue numbers on the nodes are divergence times from present, MYA. Red dots represent the calibration time. **b,** Multispecies coalescent tree of CDSs by the ASTRAL-based method. **c,** Multispecies coalescent tree of protein sequences by the ASTRAL-based method. **d,** Multispecies coalescent tree of CDSs by the MrBayes method. **e,** Ks distribution of tree peony, grape, *Arabidopsis* and wheat A genome. The Ks value is from reciprocal best gene pairs. **f,** The length distribution of gene elements of tree peony and three other species. **g,** ChIP-seq analysis of the average depth of gene and TE elements, as well as expressed and non-expressed genes in the peony intergenic region and the 2k bp external region.

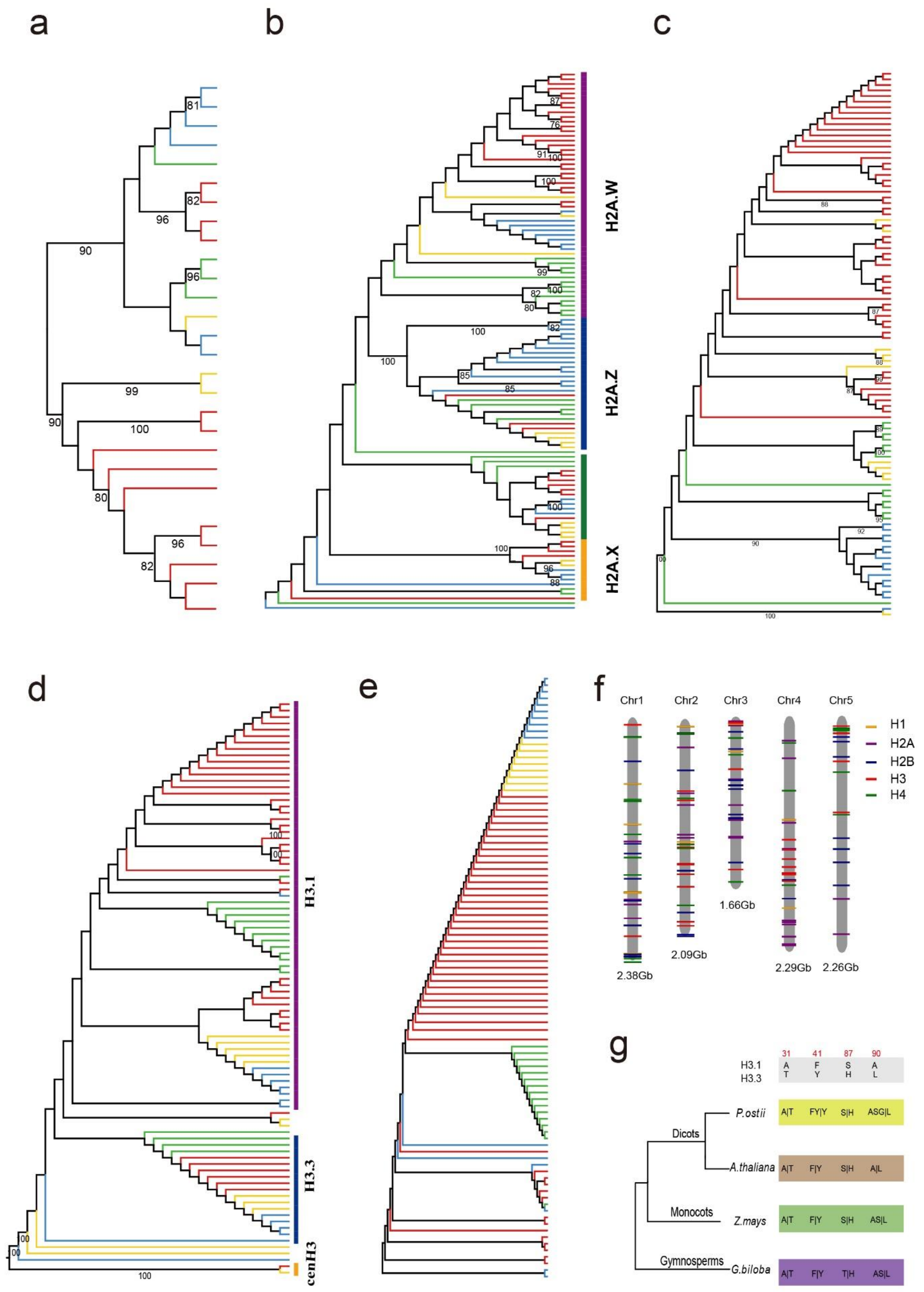
a
b
c
d
e
f
g
H2A.W
H2A.Z
H2A.X
H3.1
H3.3
cenH3
Chr1
Chr2
Chr3
Chr4
Chr5
H1
H2A
H2B
H3
H4
2.38Gb
2.09Gb
1.66Gb
2.29Gb
2.26Gb
31
41
87
90
H3.1
H3.3
Dicots
Monocots
Gymnosperms
P.ostii
A.thaliana
Z.mays
G.biloba

Supplementary Fig. 7. Genetic and genomic analysis of histones. Phylogenetic analysis of histones H1(**a**), H2A(**b**), H2B(**c**), H3(**d**), and H4(**e**). The subfamilies of H2A and H3 are annotated. **f,** distributions of histones on the five giga-chromosomes of *P. ostii.* **g,** Variation of four amino acids from H3.1 and H3.3 in *P. ostii, A. thaliana*, *Z. mays* and *G. biloba*. Source data are provided as a Source Data file.

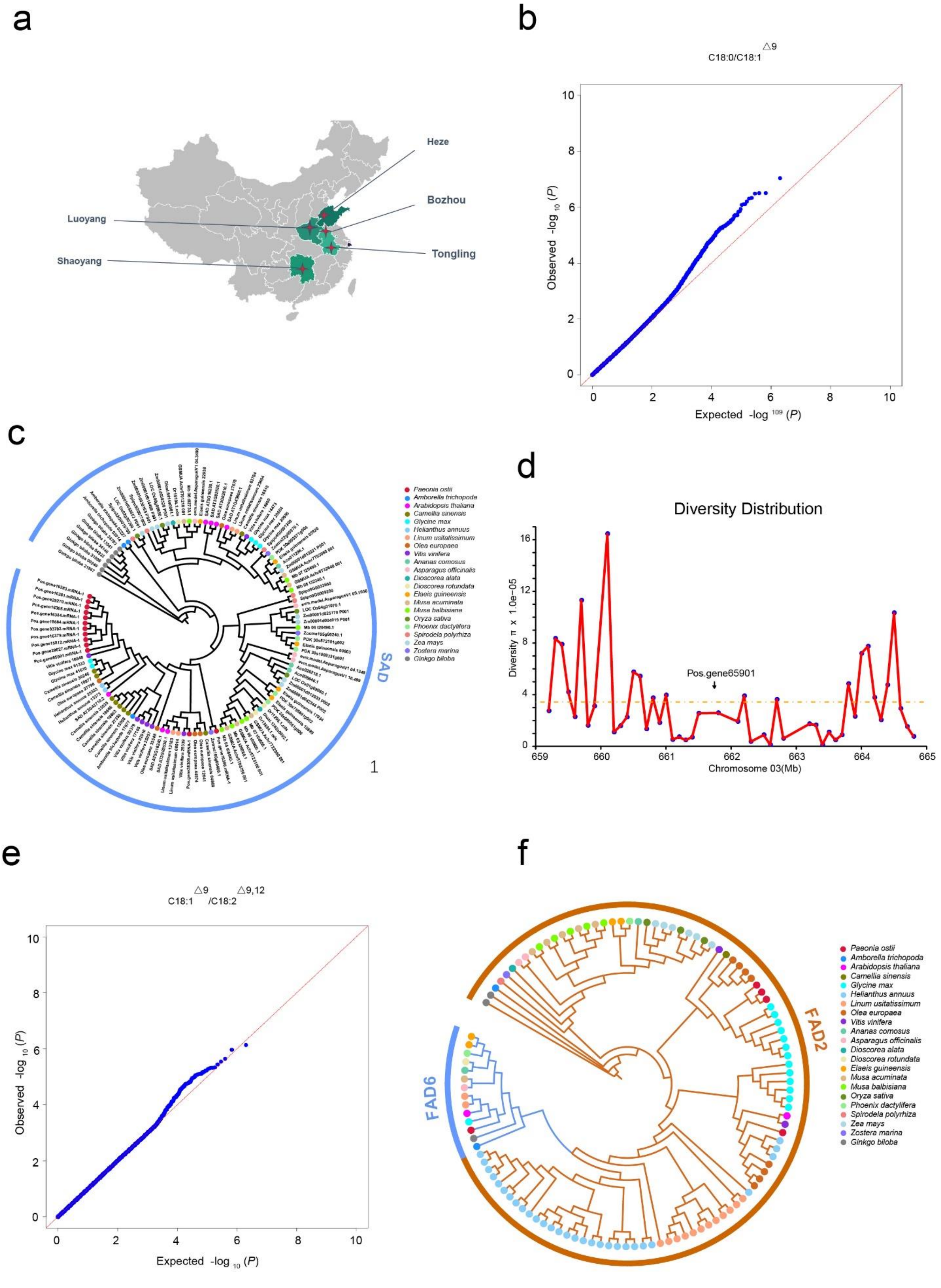
a
Heze
Bozhou
Luoyang
Tongling
Shaoyang
b
C18:0/C18:1 △9
Observed -log 10 (P)
Expected -log 109 (P)
c
SAD
Paeonia ostii
Amborella trichopoda
Arabidopsis thaliana
Camellia sinensis
Glycine max
Helianthus annuus
Linum usitatissimum
Olea europaea
Vitis vinifera
Ananas comosus
Asparagus officinalis
Dioscorea alata
Dioscorea rotundata
Elaeis guineensis
Musa acuminata
Musa balbisiana
Oryza sativa
Phoenix dactylifera
Spirodela polyrhiza
Zea mays
Zostera marina
Ginkgo biloba
1
d
Diversity Distribution
Diversity π x 1.0e−05
Pos.gene65901
Chromosome 03(Mb)
e
C18:1 △9 /C18:2 △9,12
Observed -log 10 (P)
Expected -log 10 (P)
f
FAD2
FAD6
Paeonia ostii
Amborella trichopoda
Arabidopsis thaliana
Camellia sinensis
Glycine max
Helianthus annuus
Linum usitatissimum
Olea europaea
Vitis vinifera
Ananas comosus
Asparagus officinalis
Dioscorea alata
Dioscorea rotundata
Elaeis guineensis
Musa acuminata
Musa balbisiana
Oryza sativa
Phoenix dactylifera
Spirodela polyrhiza
Zea mays
Zostera marina
Ginkgo biloba

Supplementary Fig. 8. GWAS analysis of *P. ostii* traits. a, Distribution of the collected tree peony samples. The samples were mainly collected from five locations: 'Bozhou', 'Heze', 'Luoyang', 'Shaoyang' and 'Tongling'. **b,** Quantile-quantile plot of the -log10(p) of the markers for the trait C18:0/C18:1$^{\triangle 9}$. **c,** Phylogenetic analysis of *SAD* genes in *P. ostii* and representative plants. **d,** Diversity distribution of genes in a 6 Mb region of chromosome 3. **e,** Quantile-quantile plot of the -log10(p) of the markers for the trait C18:1$^{\triangle 9}$/C18:2$^{\triangle 9,12}$. **f,** Phylogenetic analysis of *FAD2* genes in *P. ostii* and representative plants. Source data are provided as a Source Data file.

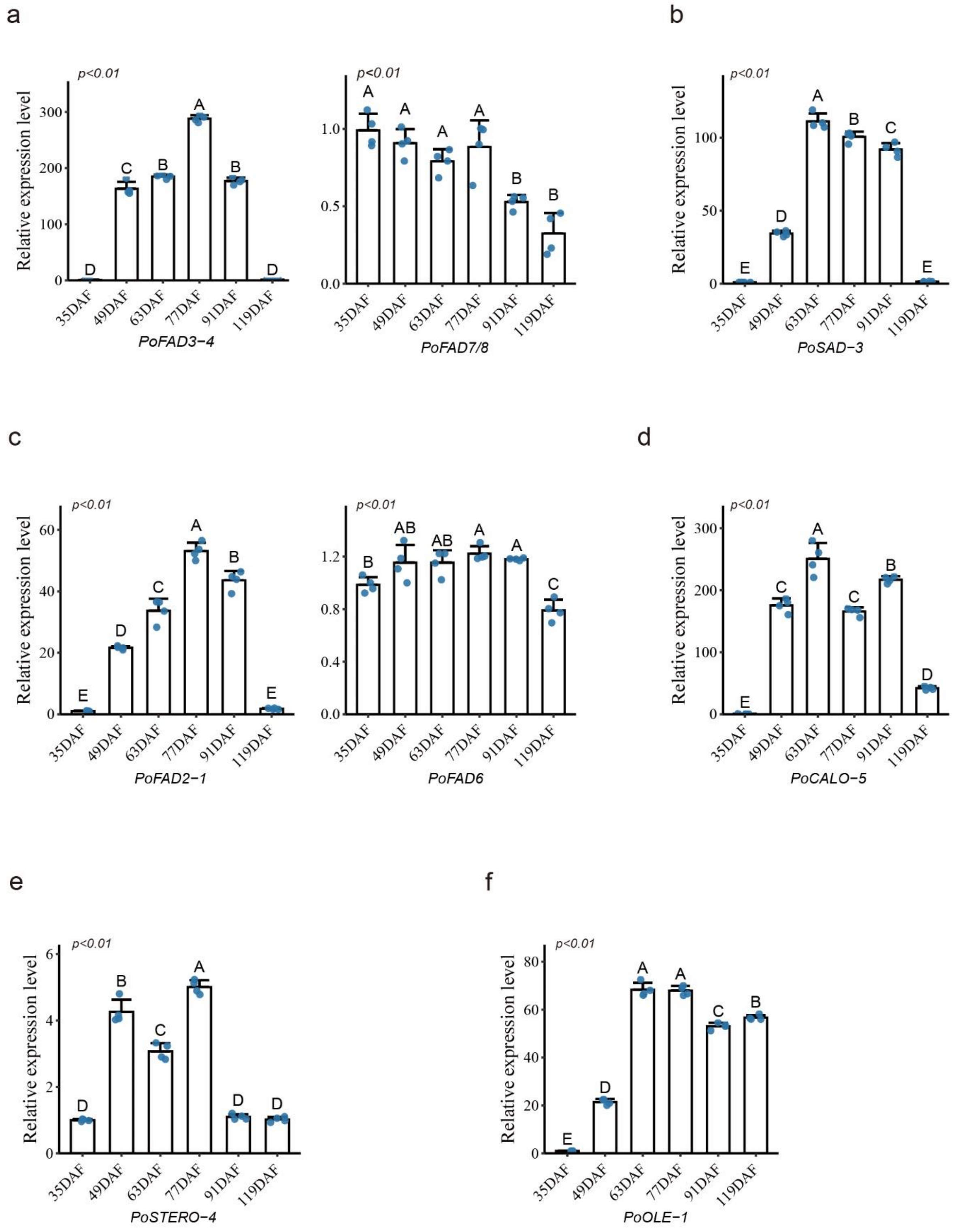

Supplementary Fig. 9. Real-time qPCR validation of the expression of fatty acid biosynthesis-related genes (a-f), at varying scales. Three replicates for each gene were performed. Data are presented as mean values +/- SEM.

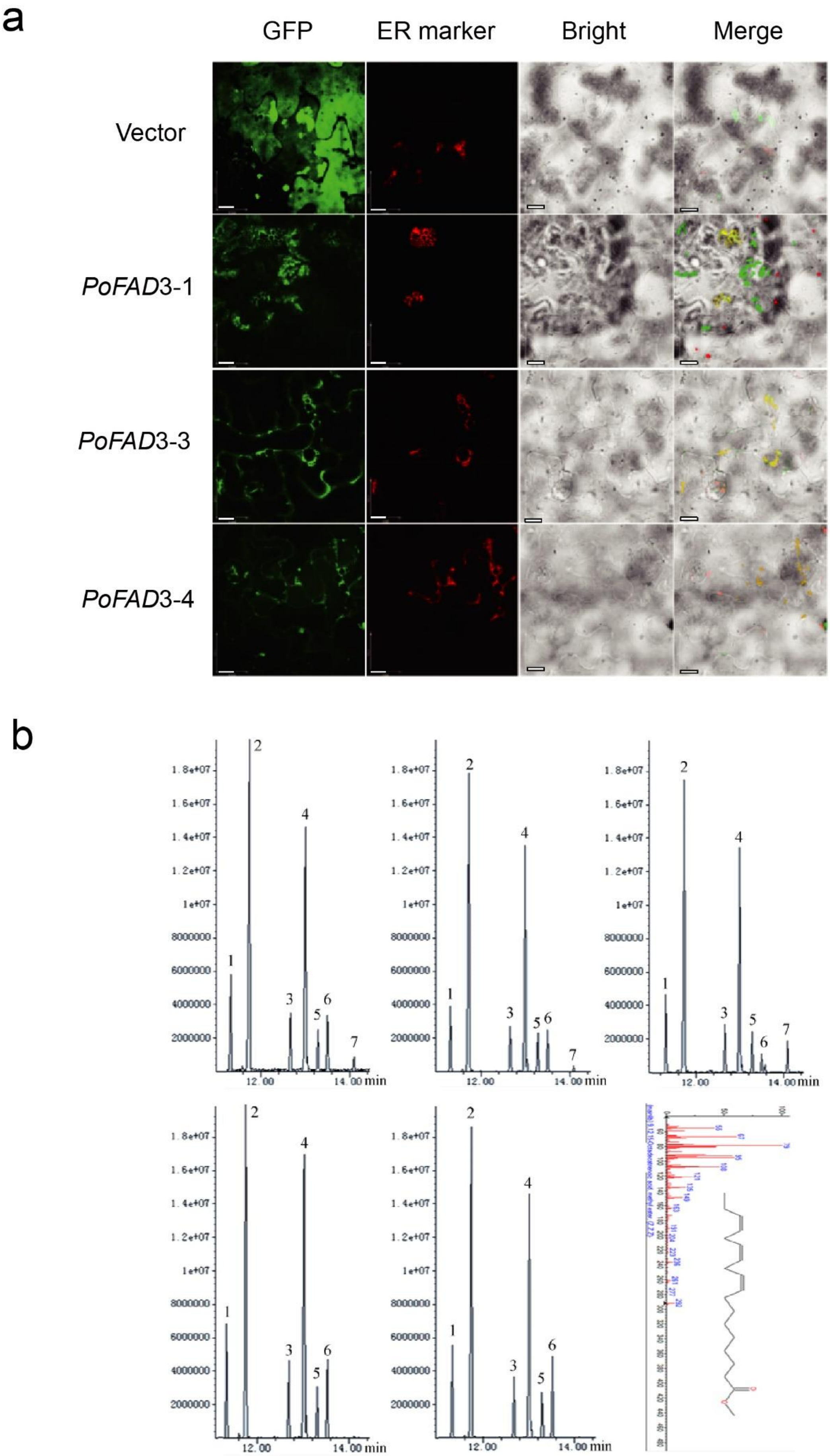
a
GFP
ER marker
Bright
Merge
Vector
PoFAD3-1
PoFAD3-3
PoFAD3-4
b
1.8e+07
1.6e+07
1.4e+07
1.2e+07
1e+07
8000000
6000000
4000000
2000000
12.00
14.00 min

Supplementary Fig. 10. Validation of omega-3 desaturase *FAD3* expression. a, Expression of GFP-tagged *FAD3* protein *in vitro*. Subnuclear localization confirmed its high expression in the ER. Scale bar for all micrographs, 10 μm. Three times of each experiment was repeated independently. **b,** GC-MS analysis of fatty acid accumulation in the yeast expression products. From top left to bottom right: transgenic yeast pY31, transgenic yeast pY33, transgenic yeast pY34, GFP-transgenic yeast pYgfp, yeast empty vector INVSc1, structure of the new product. The individual peaks are labelled as 1, C16:0, 2, C16:1, 3, C18:0, 4, C18:1$^{\triangle 9}$, 5, IS, 6, C18:2$^{\triangle 9,12}$ and 7, C18:3$^{\triangle 9,12,15}$.

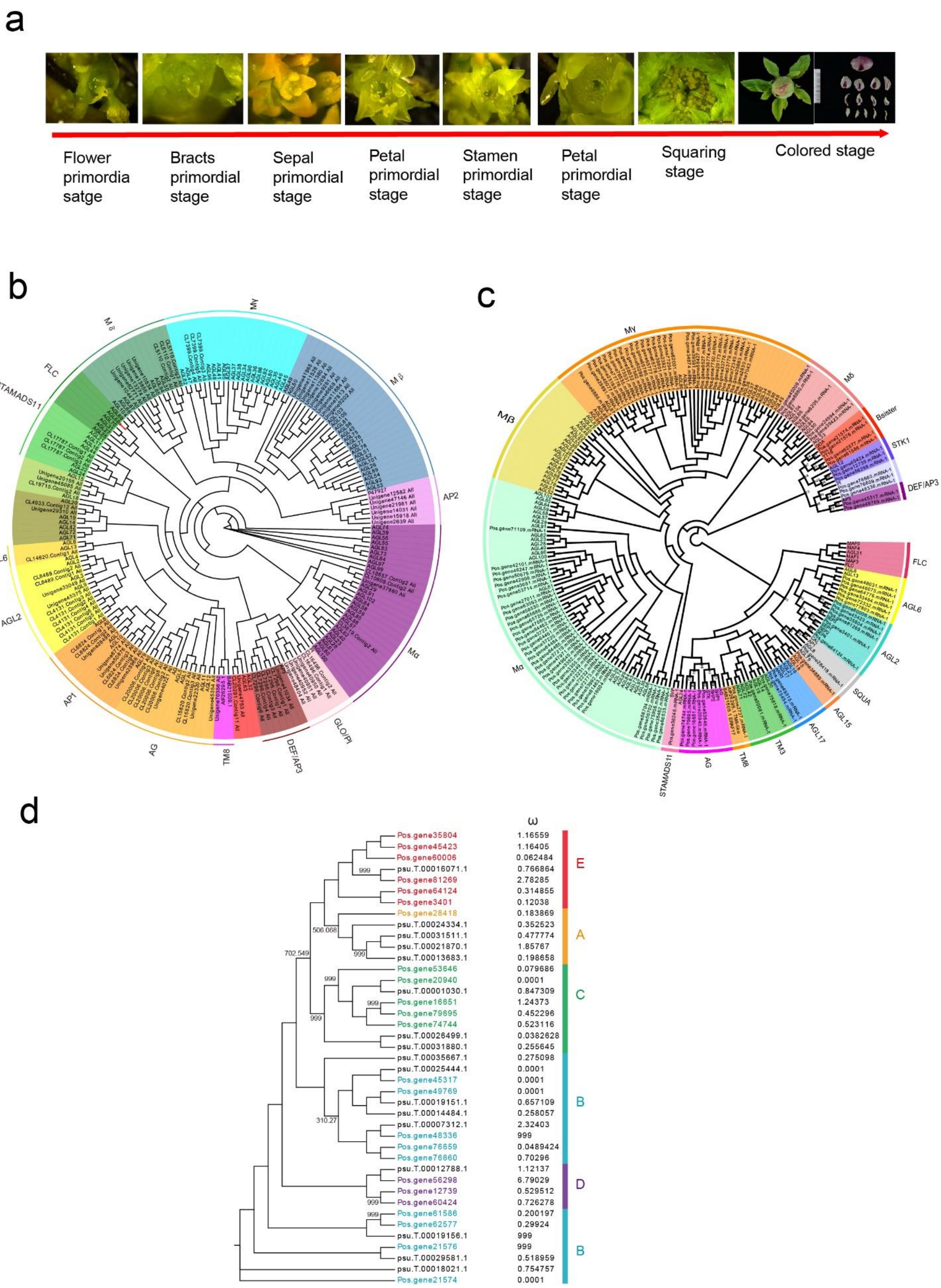
a
Flower primordia satge
Bracts primordial stage
Sepal primordial stage
Petal primordial stage
Stamen primordial stage
Petal primordial stage
Squaring stage
Colored stage
b
Mγ
Mβ
Mδ
FLC
STAMADS11
AP2
AGL6
AGL2
AP1
AG
TM8
DEF/AP3
GLO/PI
Mα
c
Mγ
Mδ
Bsister
STK1
DEF/AP3
Mβ
FLC
AGL6
AGL2
SQUA
AGL15
AGL17
TM3
TM8
AG
STAMADS11
Mα
d
ω
Pos.gene35804 1.16559
Pos.gene45423 1.16405
Pos.gene60006 0.062484
psu.T.00016071.1 0.766864
Pos.gene81269 2.78285
Pos.gene64124 0.314855
Pos.gene3401 0.12038
E
Pos.gene28418 0.183869
psu.T.00024334.1 0.352523
psu.T.00031511.1 0.477774
psu.T.00021870.1 1.85767
psu.T.00013683.1 0.198658
A
Pos.gene53646 0.079686
Pos.gene20940 0.0001
psu.T.00001030.1 0.847309
Pos.gene16651 1.24373
Pos.gene79695 0.452296
Pos.gene74744 0.523116
psu.T.00026499.1 0.0382628
psu.T.00031880.1 0.255645
C
psu.T.00035667.1 0.275098
psu.T.00025444.1 0.0001
Pos.gene45317 0.0001
Pos.gene49769 0.0001
psu.T.00019151.1 0.657109
psu.T.00014484.1 0.258057
psu.T.00007312.1 2.32403
Pos.gene48336 999
Pos.gene76659 0.0489424
Pos.gene76660 0.70296
B
psu.T.00012788.1 1.12137
Pos.gene56298 6.79029
Pos.gene12739 0.529512
Pos.gene60424 0.726278
D
Pos.gene61586 0.200197
Pos.gene62577 0.29924
psu.T.00019156.1 999
Pos.gene21576 999
psu.T.00029581.1 0.518959
psu.T.00018021.1 0.754757
Pos.gene21574 0.0001
B
999
506.068
702.549
999
999
999
999
310.27
999
999

Supplementary Fig. 11. Petaloid-stamens of *P. ostii.* **a,** Flowering differentiation and development in *P. suffruticosa.* **b,** Phylogenetic tree of genes in the ABCE model identified from *P. suffruticosa* transcriptomic data based on Bayesian methods. **c,** Phylogenetic analysis of *MADS-box* genes in *P. ostii*with RAxML methods*.* **d,** The rate of evolution of ABCE genes in *P. ostii*and *P. suffruticosa.* Source data are provided as a Source Data file.

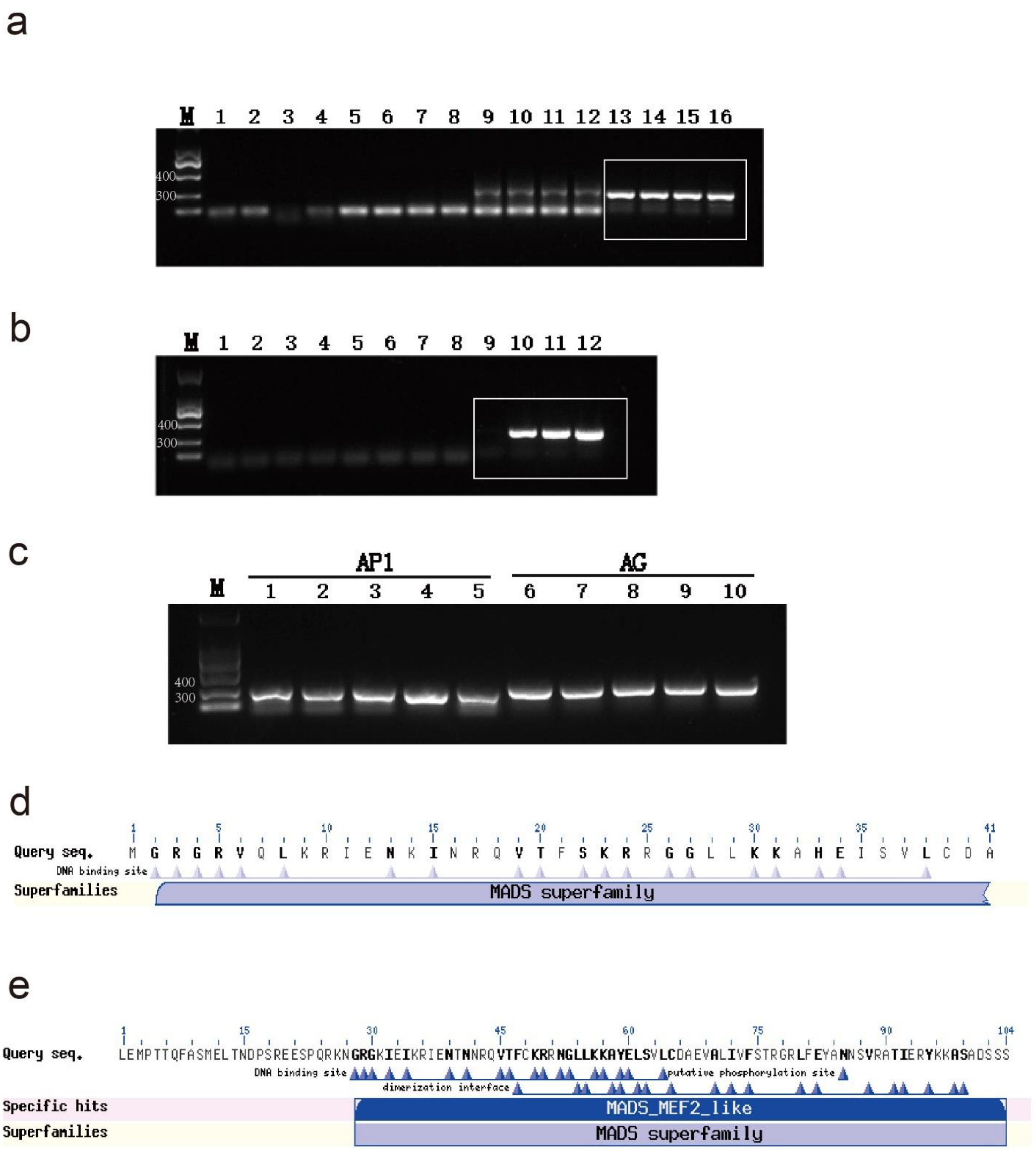

Supplementary Fig. 12. Cloning *PoAP1* and *PoAG* from *P. suffruticosa*. a, Agarose gel electrophoresis analysis of *PoAP1* (lanes 13-16). Three times of each experiment was repeated independently. **b,** Agarose gel electrophoresis analysis of *PoAG* (lanes 10-12). Three times of each experiment was repeated independently. **c,** Bacterial liquid PCR screening of positive clones of *PoAP1* and *PoAG*. Three times of each experiment was repeated independently. **d,** Domain analysis of the *PoAP1* cloning fragment. **e,** Domain analysis of the *PoAG* cloning fragment. M, DNA marker DL1000.

Supplementary Table 1. Statistics of Illumina sequencing data.

Clean reads length(bp)	Clean data(bp)	Depth(X)
100	74,390,370,000	6.19
125	257,499,996,500	21.44
125	246,291,814,500	20.51
125	165,689,612,000	13.79
230	469,803,490,302	39.12
230	451,138,298,378	37.56
49	305,980,362,212	25.48
49	398,447,818,076	33.18
49	195,335,363,902	16.26
49	241,475,980,466	20.1
49	168,412,894,650	14.02
	2,974,466,000,986	247

Supplementary Table 2. Statistics of PacBio SMRT sequencing data.

PacBio SMRT seq	Total bases (Gb)	Average read length (bp)	N50 length	Quality	Depth (X)
**RSII**	277.64	9,122	11,882	85%	23.12
**Sequel**	366.03	11,114	17,315	85%	30.48
**Total**	643.67	10,118	14,598	85%	53.6

Supplementary Table 3. Statistics of Hi-C sequencing data.

Library	Reads	Bases(Gb)
**MuDan1**	493,949,008	148.18
**MuDan14**	501,130,687	150.34
**MuDan15**	493,013,302	147.9
**MuDan2**	500,858,291	150.26
**MuDan22**	491,109,060	147.33
**MuDan22**	883,333,067	265
**MuDan22**	3,577,983,239	1073.39
**MuDan3**	499,220,075	149.77
**Total**	8,323,929,796	2497.18

Supplementary Table 4. Statistics of assembly results with only Illumina data.

	Contig		Scaffold	
	**Size(bp)**	**Number**	**Size(bp)**	**Number**
**N90**	953	3,499,042	1,719	831,674
**N80**	1,487	2,449,365	6,583	370,597
**N70**	2,139	1,744,777	14,165	212,051
**N60**	2,971	1,247,300	25,234	131,571
**N50**	3,923	880,475	40,128	83,430
**Longest**	74,358	-	859,102	-
**Total length**	12,531,002,549	-	15,273,082,638	-
**Total number(>=100bp)**	-	5,621,609	-	2,512,319
**Total number(>=2000bp)**	-	1,861,113	-	734,024
**GC rate**	0.444	-	0.291	-

Supplementary Table 5. Statistics of assembly results with only PacBio data.

	Contig		Scaffold	
	**Size(bp)**	**Number**	**Size(bp)**	**Number**
**N90**	41,010	80,832	41,010	80,832
**N80**	79,429	55,382	79,429	55,382
**N70**	116,779	40,027	116,779	40,027
**N60**	157,008	29,048	157,008	29,048
**N50**	202,017	20,692	202,017	20,692
**Longest**	2,255,483	-	2,255,483	-
**Total length**	14,887,887,589	-	14,887,887,589	-
**Total number(>=100bp)**	-	173,510	-	173,510
**Total number(>=2000bp)**	-	173,510	-	173,510
**GC rate**	0.325	-	0.325	-

Supplementary Table 6. Statistics of assembly results with Illumina data and PacBio data.

	Contig		Scaffold	
	**Size(bp)**	**Number**	**Size(bp)**	**Number**
**N90**	52,999	56,804	537,246	8,294
**N80**	95,741	39,886	794,418	6,392
**N70**	137,070	29,204	1,018,147	5,002
**N60**	181,553	21,434	1,208,328	3,870
**N50**	230,474	15,430	1,411,215	2,908
**Longest**	2,235,459	-	8,263,262	-
**Total length**	12,276,358,983	-	12,553,866,585	-
**Total number(>=100bp)**	-	117,510	-	12,779
**Total number(>=2000bp)**	-	117,206	-	12,779
**GC rate**	0.328	-	0.321	-

Supplementary Table 7. Statistics of assembly results incorporated with Hi-C data.

	Contig		Scaffold	
	**Size(bp)**	**Number**	**Size(bp)**	**Number**
**N90**	52,842	57,224	1,784,695,063	5
**N80**	95,212	40,237	1,784,695,063	5
**N70**	136,089	29,494	2,248,995,167	4
**N60**	180,049	21,667	2,432,486,241	3
**N50**	228,382	15,612	2,432,486,241	3
**Longest**	2,235,459	-	2,562,065,589	-
**Total length**	12,276,358,983	-	12,332,046,983	-
**Total number(>=100bp)**	-	117,978		6,602
**Total number(>=2000bp)**	-	117,674		6,315
**GC rate**	0.328	-	0.326	

Supplementary Table 8. Genome completion evaluation using Benchmarking Universal Single-Copy Orthologs (BUSCO) analysis.

	Complete BUSCOs	Fragmented BUSCOs	Missing BUSCOs
**Hybrid corrected scaffolds**	1298 (90.1%)	62 (4.3%)	80 (5.6%)

Supplementary Table 9. Genome completion evaluation using Illumina read mapping rates.

PE library	Lane	Mapped reads	Total reads	Mapped reads percent (%)
**180bp**	DES00030_L1	116,282,738	130,875,722	88.85
**300bp**	DES00033_L6	66,871,214	75,913,650	88.09
**500bp**	DES00015_L4	80,661,596	93,691,966	86.09
**Total**	----	263,815,548	300,481,338	87.8

Supplementary Table 10. Statistics of Illumina transcriptome sequencing data.

Sample	Total raw reads (Mb)	Total clean reads (Mb)	Total clean bases (Gb)	Clean reads Q20 (%)	Clean reads Q30 (%)	Clean reads ratio (%)
**Apical**	70.47	70.34	10.55	87.72	73.84	99.82
**Carpel**	67.78	67.61	10.14	86.95	72.93	99.75
**Leaf**	65.54	65.45	9.82	90.55	78.45	99.85
**Petal**	67.4	67.25	10.09	88.34	75.55	99.78
**Pistil**	60.93	60.79	9.12	88.34	75.64	99.76
**Seed**	74.27	74.14	11.12	87.8	74.09	99.82
**Stamen**	69.96	69.81	10.47	89.45	77.19	99.79
**Stem**	70.26	70.1	10.52	88.85	76.41	99.77

Supplementary Table 11. Genome completion evaluation using mapping coverage of the transcriptome.

Resource type	Tissue	Insert size	Number of reads pairs (Mb)	Percent aligned
**Illumina, PE**	Apical	446	76.65	78.73%
**Illumina, PE**	Carpel	502	57.56	79.45%
**Illumina, PE**	Leaf	474	49.15	80.58%
**Illumina, PE**	Petal	485	41.31	80.52%
**Illumina, PE**	Pistil	489	45.02	79.72%
**Illumina, PE**	Seed	464	68.6	78.50%
**Illumina, PE**	Stamen	471	47.43	80.14%
**Illumina, PE**	Stem	480	45.4	80.89%

Supplementary Table 12. Statistics of the five assembled chromosomes of *P. ostii*.

Chromosome	Length (bp)
**Chr01**	2,562,065,589
**Chr02**	2,248,995,167
**Chr03**	1,784,695,063
**Chr04**	2,46,5308,516
**Chr05**	2,432,486,241

Supplementary Table 13. Statistics of gene annotation of *P. ostii*.

Gene set	Number of genes	Average gene length (bp)	Average CDS length (bp)	Average exon per gene	Average exon length (bp)	Average intron length (bp)
Arabidopsis thaliana	62,725	5858.23	671.78	2.18	307.99	4390.82
Carica papaya	62,613	5726.6	555.06	2.29	241.87	3993.76
Malus x domestica	58,511	6827.31	607.62	2.49	243.71	4165.23
Kalanchoe fedtschenkoi	61,539	5735.25	560.98	2.33	241.21	3903.11
Vitis viniferea	57,794	6804.56	597.93	2.7	221.26	3645.91
**RNA-Seq**	57,566	11334.86	1707.76	4.62	369.84	2661.19
**Iso-Seq**	27,576	11484.47	1895.76	5.05	375.14	2365.54
**Final gene set**	73,177	8222.43	794.61	4.63	203.68	2001.82
**High confidence genes**	55,998	8195.43	858.22	4.71	218.4	1927.52
**Low confidence genes**	17,179	8310.4	587.26	4.37	151.91	2268.98
**High confidence genes anchored to the chromosome**	54,451	7760.1	852.17	4.65	220.54	1843.81
**Low confidence genes anchored to the chromosome**	16,244	7466.19	569.16	4.25	151.73	2100.67

Supplementary Table 14. Statistics of gene functional annotation of *P. ostii*.

		Number	Percent (%)
**Total genes anchored to chromosome**		70,695	/
	**InterPro**	53,256	75.33%
	**GO**	33,541	47.44%
**Annotated**	**KEGG**	45,399	64.22%
	**Swissprot**	46,857	66%
	**TrEMBL**	58,258	82.41%
	**Nr**	58,844	83.24%
	**KOG**	46,376	65.60%
**Total annotated**		59,768	84.54%

Supplementary Table 15. Evaluation of gene set using BUSCO single-copy orthologs.

Type	Number	Ratio
**Complete BUSCOs (C)**	1,231	85.50%
Complete and single-copy BUSCOs (S)	1,138	79.00%
Complete and duplicated BUSCOs (D)	93	6.50%
**Fragmented BUSCOs (F)**	91	6.30%
**Missing BUSCOs (M)**	118	8.20%
**Total BUSCO groups searched**	1,440	/

Supplementary Table 16. Significantly enriched KEGG pathway of *P. ostii* unique gene families (Q value < 0.01).

Pathway	Sample 11,586	Background 42,764	*P*-value	*Q*-value	Pathway ID
**Ribosome biogenesis in eukaryotes**	257	595	3.62E-23	4.52E-21	ko03008
**Ubiquitin mediated proteolysis**	332	856	3.77E-20	2.36E-18	ko04120
**ABC transporters**	206	508	2.57E-15	9.18E-14	ko02010
**Other types of *O*-glycan biosynthesis**	78	139	2.94E-15	9.18E-14	ko00514
**Ribosome**	791	2583	1.78E-12	4.46E-11	ko03010
**Isoflavonoid biosynthesis**	48	87	1.29E-09	2.68E-08	ko00943
**Flavonoid biosynthesis**	109	263	1.93E-09	3.45E-08	ko00941
**Biotin metabolism**	58	115	2.62E-09	4.09E-08	ko00780
**Fatty acid biosynthesis**	111	275	8.11E-09	1.13E-07	ko00061
**Fatty acid degradation**	99	260	1.27E-06	1.59E-05	ko00071
**Benzoxazinoid biosynthesis**	36	71	2.20E-06	2.50E-05	ko00402
**Fatty acid metabolism**	152	440	2.48E-06	2.58E-05	ko01212
**Glycolysis / Gluconeogenesis**	209	661	3.51959E-05	3.38E-04	ko00010
**Alpha-linolenic acid metabolism**	98	298	0.000938886	8.38E-03	ko00592

Supplementary Table 17. Significantly enriched KEGG pathway of *P. ostii* expanded gene families (*Q* value < 0.01).

Pathway	Sample 1,391	Background 42,764	*P*-value	*Q*-value	Pathway ID
**Pyrimidine metabolism**	96	834	7.65E-27	6.04E-25	ko00240
**RNA polymerase**	66	467	1.18E-23	4.64E-22	ko03020
**Sulfur metabolism**	29	115	5.00E-18	1.32E-16	ko00920
**Metabolic pathways**	472	10264	1.33E-17	2.62E-16	ko01100
**Tryptophan metabolism**	39	234	5.37E-17	8.48E-16	ko00380
**Photosynthesis**	68	697	1.25E-15	1.65E-14	ko00195
**Oxidative phosphorylation**	107	1501	3.69E-14	4.17E-13	ko00190
**Linoleic acid metabolism**	26	126	4.62E-14	4.56E-13	ko00591
**RNA degradation**	62	693	1.06E-12	9.30E-12	ko03018
**Caffeine metabolism**	10	17	2.03E-11	1.60E-10	ko00232
**Purine metabolism**	67	847	3.26E-11	2.34E-10	ko00230
**Protein processing in endoplasmic reticulum**	89	1390	1.35E-09	8.86E-09	ko04141
**Arachidonic acid metabolism**	16	124	2.89E-06	1.76E-05	ko00590
**DNA replication**	26	307	1.03E-05	5.80E-05	ko03030
**Fatty acid elongation**	14	118	3.11E-05	1.64E-04	ko00062
**Mismatch repair**	16	166	0.00011221	5.54E-04	ko03430
**Glyoxylate and dicarboxylate metabolism**	31	480	0.000271767	1.26E-03	ko00630

**Carbon fixation in photosynthetic organisms**	34	554	0.000360454	1.58E-03	ko00710
**N-Glycan biosynthesis**	13	135	0.000481597	2.00E-03	ko00510
**Spliceosome**	52	990	0.000564423	2.18E-03	ko03040
**Phenylalanine metabolism**	18	230	0.000580427	2.18E-03	ko00360
**Ubiquitin mediated proteolysis**	44	856	0.002165832	7.78E-03	ko04120

Supplementary Table 18. Statistics of Non-coding RNA annotation of *P. ostii*.

Type		Copy	Average length(bp)	Total length(bp)	% of genome
miRNA		122	126.51	15,434	0.000123
tRNA		3,381	74.47	251,784	0.002006
rRNA	rRNA	17,284	105.25	1,819,163	0.014491
	18S	481	1009.19	485,419	0.003867
	28S	1,005	130.05	130,701	0.001041
	5.8S	276	148.84	41,081	0.000327
	5S	15,522	74.86	1,161,962	0.009256
snRNA	snRNA	2,030	130.94	265,803	0.002117
	CD-box	724	104	75,293	0.0006
	HACA-box	94	126.7	11,910	0.000095
	splicing	1,212	147.36	178,600	0.001423

Supplementary Table 19. Comparison of gene families clustered by OrthoMCL in 13 species.

Species	Number of genes	Genes in families	Unclustered genes	Number of gene families	Unique families	Average genes per family
Paeonia ostii	54432	34798	19634	15238	3098	2.28
Arabidopsis thaliana	27342	23659	3683	12602	612	1.88
Vitis vinifera	29385	22991	6394	13747	661	1.67
Kalanchoe fedtschenkoi	30964	25386	5578	13992	528	1.81
Amborella trichopoda	25628	19183	6445	12340	869	1.55
Aquilegia coerulea	30023	24326	5697	13469	917	1.81
Carica papaya	27681	20839	6842	13529	563	1.54
Jatropha curcas	43885	37548	6337	14210	1221	2.64
Solanum lycopersicum	34726	26596	8130	13881	896	1.92
Nelumbo nucifera	40348	28743	11605	14606	1242	1.97
Actinidia chinensis	39040	27330	11710	13226	1059	2.07
Rhodiolacrenulata	31517	24225	7292	13281	775	1.82
Helianthus annuus	52213	39851	12362	14744	2148	2.7

Note: Un-clustered genes refer to special gene of current species; Unique families refer to special gene families of current species.

Supplementary Table 20. List of sequenced plant species with megagenome over 10G.

Class	Latin name	Common name	Genome size (Mb)	WGD event	Reference
**coniferophyt**	*Pinus lambertiana*	Sugar pine	31000	No information	[1]
**coniferophyt**	*Pinus taeda*	Loblolly pine	21600	No information	[2]
**coniferophyt**	*Picea abies*	Norway spruce	19700	No recent WGD	[3]
**coniferophyt**	*Abies alba*	European silver fir	17360	No information	[4]
**monocotyledon**	*Triticum aestivum*	Bread wheat, AABBDD-genome	17000	No information	[5]
**monocotyledon**	*Triticum aestivum*	Bread wheat, AABBDD-genome	17000	No information	[6]
**monocotyledon**	*Triticum aestivum*	Bread wheat, AABBDD-genome	17000	No information	[7]

**coniferophyt**	*Pseudotsuga menziesii*	Douglas-fir	16100	No information	[8]
**monocotyledon**	*Allium sativum*	Garlic	16000	Three WGD events	[9]
**monocotyledon**	*Aloe vera*	True aloe	14000	No information	[10]
**rosid**	*Paeonia suffruticosa*	Moutan peony	13800	No information	[11]
**monocotyledon**	*Triticum turgidum*	Wild emmer, AABB-genome	12000	No information	[12]
**monocotyledon**	*Triticum turgidum*	Wild emmer, AABB-genome	12000	No information	[13]
**monocotyledon**	*Triticum turgidum*	Wild emmer, AABB-genome	12000	No information	[14]
**coniferophyt**	*Ginkgo biloba*	Ginkgo tree	11750	An ancient WGD & a recent WGD	[15]
**charophyt**	*Ceratopteris richardii*	Triangle waterfern	11250	Three WGD events	[16]

Supplementary Table 21. Statistics of recently duplicated gene pairs (Ks<0.2).

Types	Sub-types	Number	Percentage
**Total gene pairs**	/	5,294	100%
**Exon number**	One single vs One multiple	347	13.11%
**Flanking sequence similarity**	Both 5' and 3' flanking similar	187	7.06%
	Only 5' flanking similar	316	11.94%
	Only 3' flanking similar	388	14.66%
	**Total**	891	33.66%
**Flanking sequence overlap with TE**	Both 5' and 3' flanking overlapped	2,515 (genes)	47.51%
	Only 5' flanking overlapped	1,054 (genes)	19.91%
	Only 3' flanking overlapped	1,011 (genes)	19.10%
	**Total**	4,580 (genes)	86.51%
**Total**	/	4,944 (genes)	93.39%

Supplementary Table 22. Significantly enriched KEGG pathway of *P. ostii* recently duplicated gene pairs with Ks<0.2 (*Q* value < 0.01).

**Pathway**	**Sample 4,710**	**Background 44,028**	***P*-value**	***Q*-value**	**Pathway ID**
**Glutathione metabolism**	78	410	3.53E-07	3.74E-05	ko00480
**Isoquinoline alkaloid biosynthesis**	31	113	5.93E-07	3.74E-05	ko00950
**Tyrosine metabolism**	50	260	3.04E-05	1.27E-03	ko00350

Supplementary Table 23. Statistics of TE content in *P. ostii*.

		Number	Length (bp)	% of repeats	% of genome
**Class I：Retroelement**					
	**LTR Retrotransposon**	5,305,122	5,397,346,348	64.08%	42.99%
	Ty1/Copia	488,008	447,924,151	5.32%	3.57%
	Ty3/Gypsy	4,736,657	4,852,220,121	57.61%	38.65%
	Others	80,457	97,202,076	1.15%	0.77%
	**Non-LTR Retrotransposon**	1,164,287	1,297,371,684	15.40%	10.33%
	LINE	1,158,633	1,289,849,060	15.31%	10.27%
	SINE	5,654	7,522,624	0.09%	0.06%
**Class II: DNA transposon**		1,339,206	1,441,833,555	17.12%	11.49%
	CMC	1,074,409	1,252,447,289	14.87%	9.98%
	Helitron	54,410	67,916,038	0.81%	0.54%
	MULE	56,381	43,574,750	0.52%	0.35%
	DNA	38,354	21,985,718	0.26%	0.18%
	hAT	35,977	21,840,392	0.26%	0.17%
	TcMar	27,456	13,694,386	0.16%	0.11%
	Others	52,219	20,374,982	0.24%	0.16%
**Tandem repeats**		1,382,437	282,282,662	3.35%	2.25%
**Unknown**		2,513	3,541,817	0.04%	0.03%
**Total repeat**		9,193,565	8,422,376,066	100.00%	67.09%

Supplementary Table 24. Estimated insertion time of LTR superfamilies.

Mya	All	Gypsy (RLG)	Copia (RLC)	Other (RLX)	All%	Gypsy (RLG)%	Copia (RLC)%	Other (RLX)%
**0**	298	194	100	6	0.002960108	0.002096663	0.01304121	0.010526316
**0.5**	6615	6001	516	99	0.065708439	0.064856044	0.067292645	0.173684211
**1**	14958	13846	951	159	0.148581532	0.14964119	0.124021909	0.278947368
**1.5**	17148	15984	1083	117	0.170335346	0.172747709	0.141236307	0.205263158
**2**	16653	15467	1167	83	0.165418388	0.167160211	0.152190923	0.145614035
**2.5**	12928	11912	986	38	0.128417038	0.128739409	0.128586333	0.066666667
**3**	10268	9419	827	26	0.101994596	0.101796213	0.107850809	0.045614035
**3.5**	7717	7025	672	19	0.076654879	0.075922964	0.087636933	0.033333333
**4**	5751	5217	515	15	0.057126113	0.056382933	0.067162233	0.026315789
**4.5**	3833	3478	344	3	0.038074142	0.037588622	0.044861763	0.005263158
**5**	2385	2116	261	3	0.023690798	0.022868753	0.034037559	0.005263158
**5.5**	1249	1101	146	2	0.012406627	0.011899101	0.019040167	0.003508772
**6**	625	559	66	0	0.00620828	0.006041414	0.008607199	0
**6.5**	221	187	33	0	0.002195248	0.00202101	0.004303599	0
**7**	23	22	1	0	0.000228465	0.000237766	0.000130412	0

Supplementary Table 25. Statistics of flanking regions of H3.1 genes overlapping with TEs.

Gene	TE type			
**Pos.gene35945**				
**Pos.gene69301**				
**Pos.gene69409**	LTR			
**Pos.gene54975**	LTR			
**Pos.gene9181**	LTR			
**Pos.gene62825**	LTR			
**Pos.gene61792**	LTR			
**Pos.gene32751**	LTR			
**Pos.gene3371**	LINE			
**Pos.gene25399**	LINE			
**Pos.gene40934**	DNA			
**Pos.gene43908**	DNA			
**Pos.gene20873**	DNA			
**Pos.gene33719**	LTR	DNA		
**Pos.gene13446**	LTR	DNA		
**Pos.gene3374**	LTR	DNA		
**Pos.gene47467**	LTR	DNA		
**Pos.gene20246**	LTR	DNA		
**Pos.gene5247**	LTR	DNA		
**Pos.gene28058**	LTR	DNA		
**Pos.gene83610**	LTR	DNA		
**Pos.gene8253**	LTR	DNA		
**Pos.gene47462**	LTR	LINE		
**Pos.gene24155**	LTR	DNA	LINE	
**Pos.gene36610**	LTR	DNA	LINE	
**Pos.gene71337**	LTR	DNA	LINE	
**Pos.gene79909**	LTR	DNA	LINE	
**Pos.gene32755**	LTR	DNA	LINE	
**Pos.gene69412**	LTR	DNA	LINE	
**Pos.gene35983**	LTR	DNA	LINE	
**Pos.gene8807**	LTR	DNA	LINE	
**Pos.gene55478**	LTR	DNA	LINE	
**Pos.gene54974**	LTR	DNA	LINE	
**Pos.gene61791**	LTR	DNA	LINE	
**Pos.gene69414**	RC	DNA	LINE	
**Pos.gene46122**	LTR	RC	LINE	
**Pos.gene30188**	LTR	DNA	LINE	RC
**Pos.gene27552**	LTR	DNA	LINE	RC

Supplementary Table 26. Statistics of *P.ostii* traits in individual years.

Trait name	Short name	2016	2017	2018	2019	Mean
**Content of alpha-linolenic acid ($C18:3^{\triangle 9,12,15}$)**	ALA	114.2±25.89	128.52±39.64	67.48±21.42	133.99±57.88	109.24±19.8
**Content of linoleic acid ($C18:2^{\triangle 9,12}$)**	LA	74.31±18.95	62.96±17.04	42.44±15.14	68.08±28.73	59.71±10.82
**Content of oleic acid ($C18:1^{\triangle 9}$)**	OA	55.62±11.41	44.25±8.43	34.33±10.79	48.14±17.34	44.21±5.96
**Content of steric acid (C18:0)**	SA	2.95±0.77	3.55±0.9	3.34±1.09	2.86±1.17	3.14±0.57
**Content of palmitic acid (C16:0)**	PA	11.71±4.09	9.81±1.62	10.59±4.19	10.62±3.47	10.42±1.48
**Content of multiple fatty acids （PA±SA±OA±LA±ALA）**	MFA	258.77±53.66	249.09±54.05	144.26±44.83	263.67±100.53	226.02±36.85
**Content of total fatty acids**	TFA	261.99±54.38	249.09±54.05	159.32±48.34	263.67±100.53	230.75±37.23
**Proportion of C18:2△9,12/C18:3△9,12,15**	LAALA	0.6616±0.1574	0.5155±0.1678	0.636±0.1228	0.5361±0.1517	0.5574±0.1249
**$C18:1^{\triangle 9}/C18:2^{\triangle 9,12}$**	C1819C182912	0.7709±0.1457	0.7368±0.1855	0.828±0.1385	0.746±0.1698	0.7717±0.1227
**$C18:0/C18:1^{\triangle 9}$**	C180C1819	0.0542±0.0137	0.083±0.0266	0.1003±0.0306	0.0637±0.0266	0.0812±0.0332
**C16:0/C18:0**	C160C180	4.07±1.42	-	3.87±6.83	-	3.51±0.91
**Content of Unsaturated fatty acids (OA±LA±ALA)**	UFA	243.97±49.57	235.74±53.03	144.26±44.83	250.2±97.44	216.12±35.89
**Content of Saturated fatty acids (PA±SA)**	SFA	14.65±4.44	13.36±2.12	13.93±4.63	13.47±4.32	13.76±2.14
**Proportion of Unsaturated fatty acids/ Saturated fatty acids**	US	17.21±2.23	17.81±3.64	10.54±2	18.66±5.71	15.83±2.3

Supplementary Table 27. Enriched pathway of differential expressed genes during endosperm maturation.

Pathway	Pathway ID	Gene number
**Alpha-linolenic acid metabolism**	ko00592	17
**Fatty acid biosynthesis**	ko00061	34
**Fatty acid metabolism**	ko00071	56
**Biosynthesis of unsaturated fatty acids**	ko01040	44
**Glycerolipid metabolism**	ko00561	50
**Glycerophospholipid metabolism**	ko00564	65
**Fatty acid elongation in mitochondria**	ko00062	3
**Inositol phosphate metabolism**	ko00562	55
**Glycosyl phosphatidylinositol(GPI)-anchor biosynthesis**	ko00563	14
**Ether lipid metabolism**	ko00565	22
**Arachidonic acid metabolism**	ko00590	11
**Sphingolipid metabolism**	ko00600	24
**Glycosphingolipid biosynthesis - globo series**	ko00603	10
**Glycosphingolipid biosynthesis - ganglio series**	ko00604	4
**Steroid biosynthesis**	ko00100	27

Supplementary Table 28. Contents of fatty acids in transgenic yeast grown at different temperature with linoleic acid (LA) added as a substrate at 72 h.

Yeast	T/°C	Fatty acid						% Conversion
		16:0	$16:1^{\Delta 9}$	18:0	$18:1^{\Delta 9}$	$18:2\Delta^{9,12}$	$18:3\Delta^{9,12,15}$	
pY31 (PoFAD3_1)	28	19.30±0.09	16.46±0.14	6.39±0.14	11.21±0.06	39.73±0.06	–	–
	25	10.29±0.06	39.28±0.22	6.88±0.02	32.33±0.21	7.04±0.48	–	–
	20	10.39±0.50	43.84±0.58	5.82±0.26	29.52±0.23	5.06±0.23	1.20±0.02	19.24±0.83
	15	10.98±0.60	49.63±0.68	3.91±0.22	27.25±0.43	3.05±0.25	2.86±0.25	48.38±3.85
	10	14.38±0.28	41.29±0.11	4.42±0.15	22.99±0.36	11.15±0.19	0.93±0.04	7.71±0.34
	5	15.02±0.17	41.72±0.04	4.23±0.06	20.16±0.01	13.34±0.22	0.92±0.03	6.47±0.11
Py33 (PoFAD3_3)	28	23.21±0.38	5.51±0.21	7.23±0.35	3.55±0.27	51.95±0.13	–	–
	25	9.93±0.05	38.49±0.16	6.25±0.18	32.52±0.29	8.30±0.28	–	–
	20	8.52±0.05	43.87±0.56	5.54±0.09	32.05±0.39	4.78±0.08	0.61±0.08	11.33±1.53
	15	11.54±0.25	49.27±1.49	3.54±0.21	28.52±0.76	2.94±0.05	1.51±0.05	34.04±0.71
	10	14.47±0.42	42.48±1.18	4.37±0.32	22.32±1.13	11.16±0.32	0.76±0.01	6.36±0.18
	5	16.43±0.19	39.13±0.37	4.55±0.28	20.97±0.12	14.10±0.19	0.46±0.04	3.16±0.25
Py34 (PoFAD3_4)	28	21.08±0.29	10.15±0.15	6.66±0.32	7.19±0.21	49.42±1.00	–	–
	25	9.89±0.17	38.14±0.73	6.45±0.34	33.02±0.38	7.03±0.30	1.31±0.03	15.73±0.65
	20	9.15±0.27	43.26±0.65	5.56±0.29	31.30±0.47	2.47±0.15	3.47±0.06	58.47±1.09
	15	12.33±0.32	48.69±0.82	3.67±0.17	27.08±0.62	2.33±0.06	3.01±0.08	56.39±0.27
	10	14.67±0.46	39.27±0.71	5.07±0.12	20.95±0.16	13.77±0.26	1.11±0.05	7.45±0.42
	5	13.41±0.98	41.86±0.16	3.97±0.24	23.38±0.21	11.97±0.65	1.26±0.08	9.51±0.13

Supplementary references

1. Stevens, K. A. *et al.* Sequence of the sugar pine megagenome. *Genetics* **204**, 1613–1626 (2016).
2. Neale, D. B. *et al.* Decoding the massive genome of loblolly pine using haploid DNA and novel assembly strategies. *Genome Biology* **15**, 1–13 (2014).
3. Nystedt, B. *et al.* The Norway spruce genome sequence and conifer genome evolution. *Nature* **497**, 579–584 (2013).
4. Mosca, E. *et al.* A reference genome sequence for the European silver fir (*Abies alba* Mill.): A community-generated genomic resource. *G3 Genes|Genomes|Genetics* **9**, 2039–2049 (2019).
5. Lukaszewski, A. J. *et al.* A chromosome-based draft sequence of the hexaploid bread wheat (*Triticum aestivum*) genome. *Science* **345**, (2014).
6. Appels, R. *et al.* Shifting the limits in wheat research and breeding using a fully annotated reference genome. *Science* **361**, (2018).
7. Walkowiak, S. *et al.* Multiple wheat genomes reveal global variation in modern breeding. *Nature* **588**, 277–283 (2020).
8. Neale, D. B. *et al.* The Douglas-Fir genome sequence reveals specialization of the photosynthetic apparatus in Pinaceae. *G3: Genes, Genomes, Genetics* **7**, 3157–3167 (2017).
9. Sun, X. *et al.* A chromosome-level genome assembly of garlic (*Allium sativum*) provides insights into genome evolution and allicin biosynthesis. *Molecular Plant* **13**, 1328–1339 (2020).

10. Jaiswal, S. K., Mahajan, S., Chakraborty, A., Kumar, S. & Sharma, V. K. The genome sequence of *Aloe vera* reveals adaptive evolution of drought tolerance mechanisms. *iScience* **24**, (2021).

11. Lv, S. *et al.* Draft genome of the famous ornamental plant *Paeonia suffruticosa*. *Ecology and Evolution* **10**, 4518–4530 (2020).

12. Avni, R. *et al.* Wild emmer genome architecture and diversity elucidate wheat evolution and domestication. *Science* **357**, 93–97 (2017).

13. Zhu, T. *et al.* Improved genome sequence of wild emmer wheat Zavitan with the aid of optical maps. *G3 Genes|Genomes|Genetics* **9**, 619–624 (2019).

14. Maccaferri, M. *et al.* Durum wheat genome highlights past domestication signatures and future improvement targets. *Nature Genetics* **51**, 885–895 (2019).

15. Guan, R. *et al.* Draft genome of the living fossil *Ginkgo biloba*. *GigaScience* **5**, 1–14 (2016).

16. Marchant, D. B. *et al.* The C-Fern (*Ceratopteris richardii*) genome: insights into plant genome evolution with the first partial homosporous fern genome assembly. *Scientific Reports* **9**, 1–14 (2019).
