# Supplementary material for: Genomic basis of the giga-chromosomes and giga-genome of tree peony Paeonia ostii
Source: Nat Commun. 2022 Nov 28;13:7328. doi: 10.1038/s41467-022-35063-1 (PMC9705720; doi:10.1038/s41467-022-35063-1)
Supplement: Supplementary file 3 — Description of Additional Supplementary Files [file 41467_2022_35063_MOESM3_ESM.pdf]

Description of Additional Supplementary Files

File Name: Supplementary Data 1
Description: Distribution of full-length LTR subtypes in land plants.

File Name: Supplementary Data 2
Description: Statistics of copy number of histones.

File Name: Supplementary Data 3
Description: Statistics of SLAF data.

File Name: Supplementary Data 4
Description: Statistics of number of SNPs and genes related to each trait.

File Name: Supplementary Data 5
Description: List of SAD and FAD2/6 genes used for phylogenetic analysis in P. ostii and representative plants.

File Name: Supplementary Data 6
Description: Candidate genes identified by GWAS in accordance to fatty acid biosynthesis related traits.

File Name: Supplementary Data 7
Description: List of genes related to fatty acid biosynthesis and the FPKM values during endosperm maturation.

File Name: Supplementary Data 8
Description: List of and FAD3 and FAD7/8 genes used for phylogenetic analysis in P. ostii and representative plants.
