# Supplementary material for: Genomic basis of the giga-chromosomes and giga-genome of tree peony Paeonia ostii
Source: Nat Commun. 2022 Nov 28;13:7328. doi: 10.1038/s41467-022-35063-1 (PMC9705720; doi:10.1038/s41467-022-35063-1)
Supplement: Supplementary file 12 — Reporting Summary [file 41467_2022_35063_MOESM12_ESM.pdf]

nature portfolio

Corresponding author(s): De-Yuan Hong, Xiao-Ya Chen, Yong-Hong Hu, Jun-hui Yuan

Last updated by author(s): Oct 10, 2022

Reporting Summary

Nature Portfolio wishes to improve the reproducibility of the work that we publish. This form provides structure for consistency and transparency in reporting. For further information on Nature Portfolio policies, see our Editorial Policies and the Editorial Policy Checklist.

Statistics

For all statistical analyses, confirm that the following items are present in the figure legend, table legend, main text, or Methods section.

n/a	Confirmed	
☐	☒	The exact sample size (*n*) for each experimental group/condition, given as a discrete number and unit of measurement
☐	☒	A statement on whether measurements were taken from distinct samples or whether the same sample was measured repeatedly
☐	☒	The statistical test(s) used AND whether they are one- or two-sided<br>*Only common tests should be described solely by name; describe more complex techniques in the Methods section.*
☐	☒	A description of all covariates tested
☐	☒	A description of any assumptions or corrections, such as tests of normality and adjustment for multiple comparisons
☐	☒	A full description of the statistical parameters including central tendency (e.g. means) or other basic estimates (e.g. regression coefficient) AND variation (e.g. standard deviation) or associated estimates of uncertainty (e.g. confidence intervals)
☐	☒	For null hypothesis testing, the test statistic (e.g. *F*, *t*, *r*) with confidence intervals, effect sizes, degrees of freedom and *P* value noted<br>*Give P values as exact values whenever suitable.*
☐	☒	For Bayesian analysis, information on the choice of priors and Markov chain Monte Carlo settings
☐	☒	For hierarchical and complex designs, identification of the appropriate level for tests and full reporting of outcomes
☒	☐	Estimates of effect sizes (e.g. Cohen's *d*, Pearson's *r*), indicating how they were calculated

Our web collection on statistics for biologists contains articles on many of the points above.

Software and code

Policy information about availability of computer code

Data collection	No software was used.
Data analysis	SOAPnuke (v1.5.5) GenomeScope (v.1.0) SOAPdenovo (v2.04) WTDBG (v1.2.8) BLASR (v5.1) BWA (v0.7.17) PILON (v1.23) SSPACE (v2.1.1) HISAT2 (v2.0.0-beta) BUSCO (v3.0.2) JUICER (v1.5) 3D-DNA (v180922) JuiceBox (v1.11.08) LTR_FINDER (v1.0.6) LTRharvest (v1.5.9) LTRdigest (v1.5.9) RepeatModeler (v1.0.8) RepeatMasker (v4.0.6) RepeatProteinMask (v.4.0.6) TRF (v4.04) Exonerate (v2.2.0) Augustus (v3.2.1) SNAP (v2006-07-28) StringTie (v1.2.1) PASA_lite (v0.1.0) Quiver (v2.3.2) Proovread (v2.14.1) GMAP (v2017-11-15) MAKER (v2.31.8) InterProScan (v5.11-51.0) tRNAscan-SE-1.23 (v1.3.1) INFERNAL (v1.1.2) OrthoMCL (v2.0.9) MUSCLE (v3.8.31) trimAl (v1.4) CAFÉ (v2.1) PAML (v4.8) CLUSTALW (v2.1) MEGA (v7.0) SHAPEIT (v4.0) PHYLIP (v3.696) GCTA (v1.91.7beta) ADMIXTURE (v1.3.0) TASSEL (v5.0) GAPIT (v3.0) LDBlockshow (v1.36)

For manuscripts utilizing custom algorithms or software that are central to the research but not yet described in published literature, software must be made available to editors and reviewers. We strongly encourage code deposition in a community repository (e.g. GitHub). See the Nature Portfolio guidelines for submitting code & software for further information.

Data

Policy information about availability of data

All manuscripts must include a data availability statement. This statement should provide the following information, where applicable:

- Accession codes, unique identifiers, or web links for publicly available datasets
- A description of any restrictions on data availability
- For clinical datasets or third party data, please ensure that the statement adheres to our policy

The original data are publicly available from China National Gene Bank with accession number CNP0003098. (https://db.cngb.org/search/project/CNP0003098/). Several public databases were used in this study, including Repbase database (v21.01), KEGG (https://www.kegg.jp/), SwissProt/TrEMBL (https://www.uniprot.org/), GO (http://www.geneontology.org/), InterPro (https://www.ebi.ac.uk/interpro/) database, SwissProt/TrEMBL (https://www.uniprot.org/), NR databases (https://www.ncbi.nlm.nih.gov/refseq/about/nonredundantproteins/) and Rfam database (Release 9.1).

Human research participants

Policy information about studies involving human research participants and Sex and Gender in Research.

Reporting on sex and gender	N/A
Population characteristics	N/A
Recruitment	N/A
Ethics oversight	N/A

Note that full information on the approval of the study protocol must also be provided in the manuscript.

Field-specific reporting

Please select the one below that is the best fit for your research. If you are not sure, read the appropriate sections before making your selection.

☐ Life sciences ☐ Behavioural & social sciences ☒ Ecological, evolutionary & environmental sciences

For a reference copy of the document with all sections, see nature.com/documents/nr-reporting-summary-flat.pdf

Ecological, evolutionary & environmental sciences study design

All studies must disclose on these points even when the disclosure is negative.

Study description	Sampling for the population analysis of 448 individuals of P. ostii
Research sample	Tree peony from five areas including Luoyang, Tongling, Bozhuo, Hezi and Shaoyang of China were introduced in the special Paeoniaceae nursery at Shanghai Chenshan Botanical Garden (31°4′52″N, 121°10′14″E) since 2014. As these individual plants were originally from various locations, they were kept in Shanghai Chenshan Botanical Garden for universal growth conditions to ensure the consistency of samples. 448 samples were proper numbers for further GWAS analysis with statistical significance.
Sampling strategy	The budded flowers of each of 448 plants were hand-pollinated with the pollen collected from the same P. ostii plant. The seeds were obtained per plant annually. No sample size calculation was performed. In most of previous studies with GWAS analysis, approximately 300 samples were sufficient for statistical analysis. 448 samples were proper numbers for GWAS with general acceptance of the researchers in this area.
Data collection	Professor Junhui Yuan, lab members Mingyu Liu, Juan Li, Lihong Lin, Linjuan Zhang, Ying Zhang and several workers paid the hour in the botanic garden conducted data collection. They counted the number of seeds, the phenotypes of the plants and recorded the data with pen and papers.
Timing and spatial scale	Over four growing seasons from 2016 to 2019. Four continuous years sampling were proper for the biological replicates of the experimental design.
Data exclusions	No data was excluded.
Reproducibility	The replicates were stated in each individual method descriptions. In most of the scenarios, three samples were used for replication. All attempts at replication were successful.
Randomization	The samples were randomly selected in the botanical garden. The allocation was random in this study.

Blinding	NA. The samples were randomly selected in the botanical garden. No specific tasks were given to the workers who collected the samples.

Did the study involve field work? ☒ Yes ☐ No

Field work, collection and transport

Field conditions	The environment is with an average of 17.6 degrees in temperature and 1173.4mm of rainfall. Shanghai is in the Eastern part of China, in the temperate zone.
Location	Shanghai Chenshan Botanical Garden (31°4′52″N, 121°10′14″E)
Access & import/export	The workers have authorized access to the local botanical garden. No laws was applicable for sample collection in the local botanical garden.
Disturbance	No disturbance was applied during our field work, collection and transport in the local botanical garden.

Reporting for specific materials, systems and methods

We require information from authors about some types of materials, experimental systems and methods used in many studies. Here, indicate whether each material, system or method listed is relevant to your study. If you are not sure if a list item applies to your research, read the appropriate section before selecting a response.

Materials & experimental systems

n/a	Involved in the study
☐	☒ Antibodies
☒	☐ Eukaryotic cell lines
☒	☐ Palaeontology and archaeology
☒	☐ Animals and other organisms
☒	☐ Clinical data
☒	☐ Dual use research of concern

Methods

n/a	Involved in the study
☐	☒ ChIP-seq
☒	☐ Flow cytometry
☒	☐ MRI-based neuroimaging

Antibodies

Antibodies used	anti-histone H3 antibody (tri methyl K27) by Cloud-Seq Biotech (Shanghai, China)
Validation	Rabbit polyclonal to Histone H3 (tri methyl K27) - ChIP Grade

ChIP-seq

Data deposition

☒ Confirm that both raw and final processed data have been deposited in a public database such as GEO.

☒ Confirm that you have deposited or provided access to graph files (e.g. BED files) for the called peaks.

Data access links<br>*May remain private before publication.*	The original data are publicly available from China National Gene Bank with accession number CNP0003098. (https://db.cngb.org/search/project/CNP0003098/)
Files in database submission	Original reads data generated by CHIP-seq.
Genome browser session<br>(e.g. UCSC)	no longer applicable.

Methodology

Replicates	Three replicates
Sequencing depth	high-throughput 150 bp paired-end sequencing on Illumina Hiseq sequencer
Antibodies	anti-histone H3 antibody (tri methyl K27) by Cloud-Seq Biotech (Shanghai, China)

Peak calling parameters	-t for treatment file (ChIP tags, this is the ONLY required parameter for MACS) and -c for control file containing mapped tags; --format for input file format in BED or ELAND (output) format (default BED); --name for name of the run (for example, FoxA1, default NA); --gsize for mappable genome size to calculate λBG from tag count (default 2.7G bp, approximately the mappable human genome size); --tsize for tag size (default 25); --bw for bandwidth, which is half of the estimated sonication size (default 300); --pvalue for p-value cutoff to call peaks (default 1e-5); --mfold for high-confidence fold-enrichment to find model peaks for MACS modeling (default 32); --diag for generating the table to evaluate sequence saturation (default off).
Data quality	Overlapping enriched peaks are merged, and each tag position is extended d bases from its center. The location with the highest fragment pileup, hereafter referred to as the summit, is predicted as the precise binding location.
Software	MACS (Model-based Analysis of ChIP-Seq) was used for data analysis.
