# Supplementary material for: Genomic basis of the giga-chromosomes and giga-genome of tree peony Paeonia ostii
Source: Nat Commun. 2022 Nov 28;13:7328. doi: 10.1038/s41467-022-35063-1 (PMC9705720; doi:10.1038/s41467-022-35063-1)
Supplement: Supplementary file 13 — Source Data [file 41467_2022_35063_MOESM13_ESM.zip › Source Data Figure 3a.Evolutionary scenario of chromosome rearrangements and structural evolution from the eudicot ancestor to peony and other plant genomes..docx]

1. **Chromosome rearrangements from the Eudicot ancestor to tree peony**

Using *V. vinifera* as the reference genome, we first identified the homologous gene pairs using blastp (e value<1e-5) and retained only the top 10 hits for each gene (the blastp result containing 194,159 paralog gene pairs). Then, the syntenic blocks of *V. vinifera* genome were detected by MCScanX (<http://chibba.pgml.uga.edu/mcscan2/>; parameters: -e 1e-5 -k 50 -g -1 -s 5) based on the identified homologous gene pairs. We constructed the eudicot’s seven ancestral chromosomes according to the grape genome synteny (Jaillon *et al.,* 2007), the seven ancestral chromosomes contain 7,343 genes. Based on the constructed seven ancestral chromosomes, we detected the syntenic blocks between tree peony and ancestor, and we found 171 blocks between them with an average gene number of 19 per blocks. The block average lengths were 808kb in ancestral chromosomes and 24Mb in peony (Table 1). The method is same as previously mentioned, using MCscanX to detect synteny based on orthologous gene pairs identified by blastp (Parameters: -e 1e-5 -k 50 -g -1 -s 5). We further detected synteny between seven ancestral chromosomes and other plant species: *P. persica*, *V. vinifera,* and *A. thaliana* using the same method (Table 1).

Finally, based on the synteny analysis, we displayed chromosomes rearrangement and structural evolution from the ancestral chromosomes to present species and their phylogenetic relationship. The results were shown as Figure 1.

Table 1: The statistics of syntenic blocks between ancestral chromosomes and other species.

| **Query** | **Subject** | **Number of Blocks** | **Average Gene Pairs Per Syntenic Block** | **Total Syntenic Gene Pairs** | **Average blocks length(bp) in query** | **Average blocks length(bp) in subject** |
| --- | --- | --- | --- | --- | --- | --- |
| *V. vinifera* | *V. vinifera* | 159 | 14.42 | 2,292 | 1,041,999 | / |
| Ancestor | Peony | 171 | 19.11 | 3,267 | 808,130 | 23,992,527 |
| Ancestor | *V. vinifera* | 120 | 65.93 | 7,912 | 2,119,228 | 2,054,725 |
| Ancestor | *P. persica* | 222 | 24.48 | 5,434 | 940,003 | 453,681 |
| Ancestor | *A. thaliana* | 348 | 14.26 | 4,964 | 1,083,987 | 147,593 |


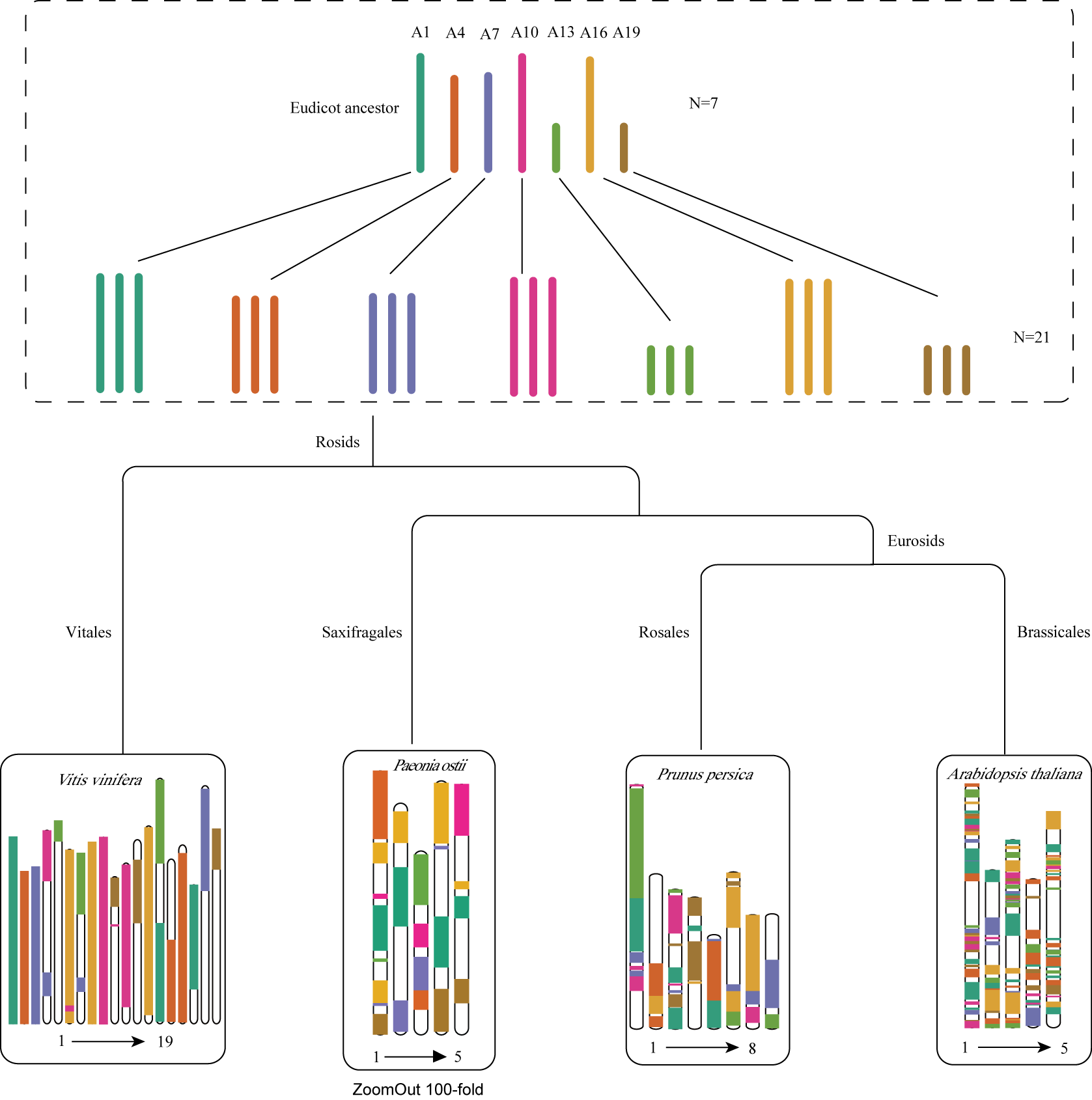


Figure 1. Evolutionary history of genome rearrangements from the eudicot ancestor to tree peony (the chromosome length was zoom out 100-fold) and other plant genomes. The eudicot ancestor chromosomes are represented with a seven-color code on top and constructed from grape genome.
